# The epidemiology of *Plasmodium vivax* among adults in the Democratic Republic of the Congo

**DOI:** 10.1038/s41467-021-24216-3

**Published:** 2021-07-07

**Authors:** Nicholas F. Brazeau, Cedar L. Mitchell, Andrew P. Morgan, Molly Deutsch-Feldman, Oliver John Watson, Kyaw L. Thwai, Pere Gelabert, Lucy van Dorp, Corinna Y. Keeler, Andreea Waltmann, Michael Emch, Valerie Gartner, Ben Redelings, Gregory A. Wray, Melchior K. Mwandagalirwa, Antoinette K. Tshefu, Joris L. Likwela, Jessie K. Edwards, Robert Verity, Jonathan B. Parr, Steven R. Meshnick, Jonathan J. Juliano

**Affiliations:** 1 Department of Epidemiology, Gillings School of Global Public Health, University of North Carolina, Chapel Hill, NC USA; 2 Medical Scientist Training Program, School of Medicine, University of North Carolina, Chapel Hill, NC USA; 3 Department of Bioinformatics & Computational Biology, University of North Carolina, Chapel Hill, NC USA; 4 Medical Research Council Centre for Outbreak Analysis and Modelling, Department of Infectious Disease Epidemiology, Imperial College London, London, UK; 5 UCL Genetics Institute, University College London, London, UK; 6 Department of Evolutionary Anthropology, University of Vienna, Vienna, Austria; 7 Institute for Global Health and Infectious Diseases, School of Medicine, University of North Carolina, Chapel Hill, NC USA; 8 Department of Geography, University of North Carolina, Chapel Hill, NC USA; 9 Department of Biology, Duke University, Durham, NC USA; 10 Duke Center for Genomic and Computational Biology, Durham, NC USA; 11 Kinshasa School of Public Health, Kinshasa, Democratic Republic of the Congo; 12 Programme National de la Lutte Contre le Paludisme, Kinshasa, Democratic Republic of Congo; 13 Division of Infectious Diseases, School of Medicine, University of North Carolina, Chapel Hill, NC USA; 14 Curriculum in Genetics and Molecular Biology, School of Medicine, University of North Carolina, Chapel Hill, NC USA

**Keywords:** Phylogenetics, Malaria, Epidemiology

## Abstract

Reports of *P. vivax* infections among Duffy-negative hosts have accumulated throughout sub-Saharan Africa. Despite this growing body of evidence, no nationally representative epidemiological surveys of *P. vivax* in sub-Saharan Africa have been performed. To overcome this gap in knowledge, we screened over 17,000 adults in the Democratic Republic of the Congo (DRC) for *P. vivax* using samples from the 2013-2014 Demographic Health Survey. Overall, we found a 2.97% (95% CI: 2.28%, 3.65%) prevalence of *P. vivax* infections across the DRC. Infections were associated with few risk-factors and demonstrated a relatively flat distribution of prevalence across space with focal regions of relatively higher prevalence in the north and northeast. Mitochondrial genomes suggested that DRC *P. vivax* were distinct from circulating non-human ape strains and an ancestral European *P. vivax* strain, and instead may be part of a separate contemporary clade. Our findings suggest *P. vivax* is diffusely spread across the DRC at a low prevalence, which may be associated with long-term carriage of low parasitemia, frequent relapses, or a general pool of infections with limited forward propagation.

## Abstract (web summary)

Plasmodium vivax generally accounts for a low proportion of malaria cases in Africa, but population-level data on the distribution of infections is limited. Here, the authors use data from the Democratic Republic of the Congo and show that the prevalence is low (~3%) and diffusely spread.

## Introduction

*Plasmodium vivax* is the most prevalent malaria-causing parasite in many locations outside of Africa, accounting for ~6.4 million cases in 2019^1^.The relative absence of *P. vivax* in Africa has long been attributed to the high prevalence of the Duffy-negative phenotype throughout most of sub-Saharan Africa (SSA)^2–4^. However, recent evidence has demonstrated that *P. vivax* infections are occurring throughout SSA among both Duffy-positive and Duffy-negative hosts^5^. Although these *P. vivax* infections have been associated with clinical cases, the distribution and extent of asymptomatic versus symptomatic disease in SSA remains unclear^5–7^.

Despite growing concern, no studies have systematically evaluated the burden, risk factors, spatial distribution, or origins of these SSA *P. vivax* infections. This lack of research is problematic as resources have begun to be directed towards diagnosing and addressing SSA *P. vivax*. If *P. vivax* were returning or reemerging in SSA as a new epidemic, it would have the potential to undermine years of malaria control and elimination efforts. To address this critical gap in knowledge, we used samples from the Democratic Republic of the Congo (DRC) 2013–2014 Demographic Health Survey (DHS) to screen a nationally representative population of over 17,000 adults for *P. vivax*. Surveys from the DHS program are community-based and are expected to contain mostly healthy, asymptomatic participants. The DRC is situated in the center of SSA and is the largest country by geographic size. Moreover, previous work has indicated that the DRC is a watershed region that links East and West Africa malaria^8,9^. As a result, findings from the DRC are highly relevant for contextualizing vivax malaria in SSA.

Using this nationally representative survey, we provide a national level estimate of *P. vivax* prevalence, associated risk factors, and the geographical distribution of cases across the DRC. In addition, we use mitochondrial genomes to identify the potential origins of these infections. By coupling a nationally representative, spatially rich dataset with robust risk factor and spatial analysis, we advance efforts to uncover the hidden distribution of *P. vivax* in SSA.

## Results

### Study population and molecular validation

**Fig. 1:**
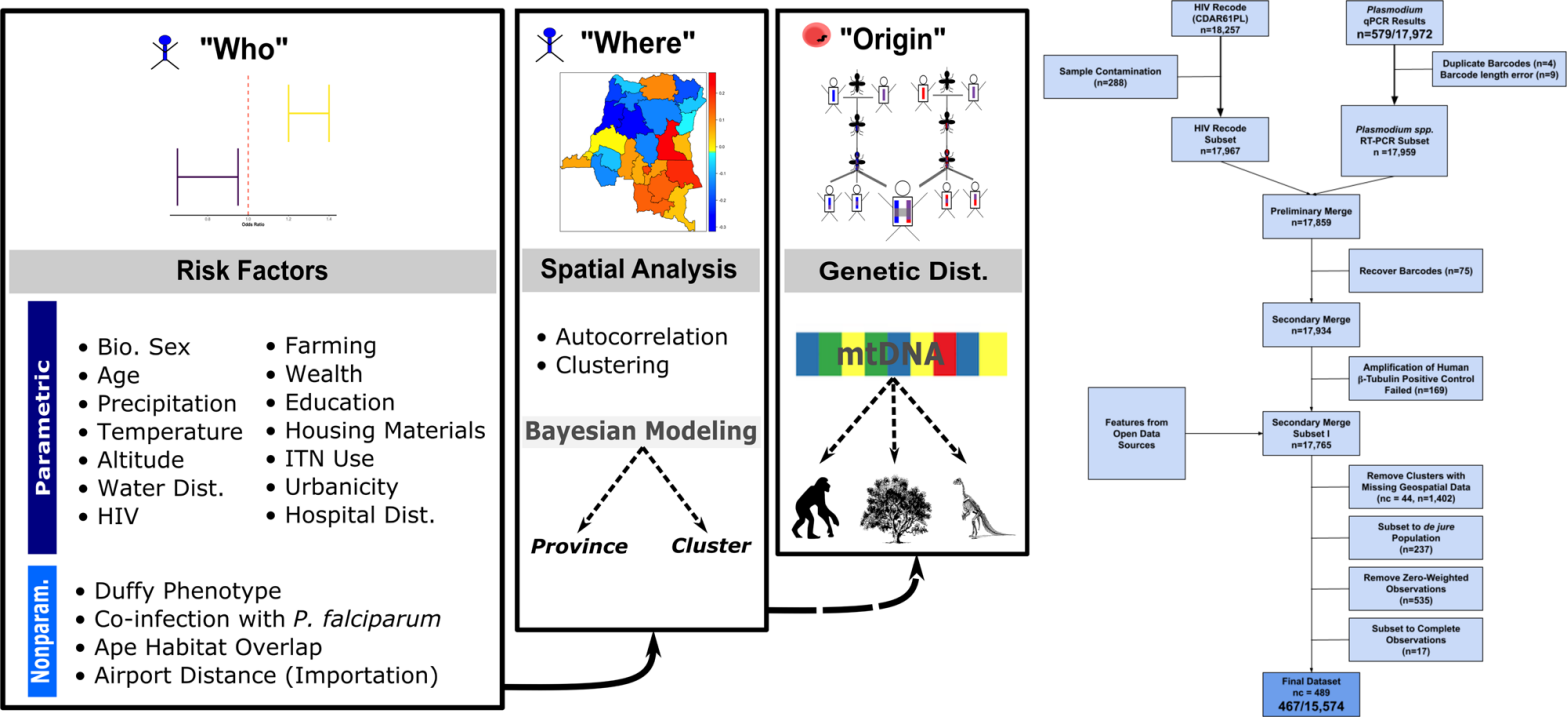
Overview of study approach and inclusion. The conceptual diagram (*left*) describes the study approach to identify “who” was being infected by *P. vivax*, “where” *P. vivax* infections were occurring, and the potential source, or “origin”, of these infections. Risk factors identified in the “who” analysis, were considered in the spatial models. Genetic distance analyses were restricted to the mitochondrial genome (mtDNA) and few DRC samples (*n* = 3). As a result, we limited this analysis to three broad questions: are the DRC samples most similar to (1) non-human apes mitochondrial strains; (2) contemporary *P. vivax* mitochondrial strains; or (3) an ancestral *P. vivax* mitochondrial strain. The final data set (*right*) consisted of 467 *P. vivax* infections among 15,574 individuals across 489 clusters, prior to the application of the Demographic Health Survey (DHS) sampling weights. Specifically, of the 18,257 Demographic Health Survey (DHS) records that had a dried blood spot, 17,972 samples were successfully shipped to the University of North Carolina for processing. Of these 17,972 samples, a small portion were lost due to barcoding errors, while the remaining 17,934 (99.79%) were successfully linked to the 2013–2014 DRC DHS survey. Of these 17,934 samples, 169 samples failed to amplify human beta-tubulin (i.e. positive control), 1402 individuals had missing geospatial data, 484 individuals were classified as de facto (visitors rather than household members), 535 individuals had zero-weighted DHS sampling weights, and 17 individuals had incomplete data and were excluded from analysis (further details in Supplementary Materials: Study Population and Data Sources). In total, our final dataset consisted of 15,574 individuals across 489 clusters. Similarly, although we identified a total of 579 *P. vivax* infections, 112 infections were lost during the aforementioned processing steps, which resulted in a final, unweighted count of 467 *P. vivax* infections.

*P. vivax* infections were first identified using quantitative PCR (qPCR) and then confirmed with a nested-PCR assay. Our *P. vivax* qPCR assay achieved an analytical sensitivity of 94% and analytical specificity of 100% (zero false positive calls) when at least 1.25 × 10^−7^ ng/μL of 18S rRNA plasmid (equivalent to approximately the number of copies of 18S rRNA in 6 genomes/μL) was present. No off-target amplification was observed when the qPCR assay was challenged with highly concentrated DNA templates from other *Plasmodium* species (Supplementary Fig. 1, Supplementary Table 2). Of the 17,972 samples that underwent screening for *P. vivax* infection, 579 were positive by qPCR. Among the 579 *P. vivax* qPCR-positive samples, 534 were confirmed by nested-PCR (92.22%), with strong agreement between the initial and reflex confirmatory assays (Cohen’s ${\mathscr{K}}$= 0.80, *p* < 0.05). All samples selected for Duffy-Genotyping validation had concordant HRM-qPCR and Sanger sequencing results, except for one sample that failed genotyping (Supplementary Text: Duffy-Genotyping). We restricted our prevalence estimates to the 467 *P. vivax* infections that were confirmed by both qPCR and reflex nested-PCR (*n*_weighted_: 459.18, 95% CI_weighted_: 346.54, 571.82) and were among the 15,574 adults included in our study (*n*_weighted_: 15,490.20, 95% CI_weighted_: 14,060.60, 16,919.80; Fig. 1).

### Prevalence of *P. vivax* and descriptive statistics among adults in the DRC

The national weighted prevalence of *P. vivax* among adults was 2.96% (95% CI_weighted_: 2.28, 3.65%). Among the 489 clusters considered, 172 clusters were positive for *P. vivax* (range: 1–9 infections per cluster) with 64 clusters containing a single infection. When the DHS sampling weights were applied, *P. vivax* infection counts ranged from 0.15 to 30.63 among the 172 clusters. Given that DHS sampling weights are applied at the cluster-level, *P. vivax* unweighted and weighted prevalences were the same and ranged from 0 to 46.15% with wide confidence intervals (Supplementary Fig. 2).

In contrast, we identified 5179 *P. falciparum* infections (*n*_weighted_: 4651.94, 95% CI_weighted_: 4121.93, 5181.94) accounting for a weighted national prevalence of 30.03% (95% CI_weighted_: 27.87, 32.19%). Overall, we identified 174 *P. falciparum*–*P. vivax* co-infections that were reduced to 145.29 co-infections when DHS sampling weights were applied (95% CI_weighted_: 108.11, 182.48; Table 1). Individual characteristics differed by infection status and suggested differences in demographic and behavioral factors between those individuals infected with *P. vivax* or *P. falciparum* versus those not infected (Table 1).

**Table 1:** Descriptive statistics of identified risk factors among individuals with *P. vivax* infections, *P. falciparum* infections, and those that are uninfected with DHS sampling weights applied: risk factor distributions appeared to differ by infection status.

Covariate	*P. vivax* infection	*P. falciparum* infection	Uninfected
N*	459.18	4651.94	10,524.38
Vivax–Falciparum co-infection	145.29		–
Urban (%)	195.75 (42.63)	1573.16 (33.82)	4445.27 (42.24)
Distance to healthcare facilities (far, %)	247.34 (53.86)	2524.99 (54.28)	4645.82 (44.14)
HIV (positive, %)	8.09 (1.76)	26.76 (0.58)	117.99 (1.12)
Biological sex (male, %)	235.89 (51.37)	2435.38 (52.35)	4790.0 (45.51)
Farmer (%)	202.64 (44.13)	2061.67 (44.32)	4181.96 (39.74)
Housing materials (traditional, %)	262.10 (57.08)	3127.13 (67.22)	5555.21 (52.78)
Education (lower, %)	181.79 (39.59)	1975.86 (42.47)	4063.91 (38.61)
ITN use (no, %)	213.83 (46.57)	2611.93 (56.15)	5301.37 (50.37)
Wealth (poor, %)	168.12 (36.61)	2209.84 (47.50)	4210.55 (40.01)
Precipitation (mm, SD)	131.97 (25.79)	137.85 (24.97)	138.33 (24.70)
Temperature (C, SD)	25.98 (1.99)	26.75 (1.35)	26.15 (1.80)
Altitude (m, SD)	771.46 (489.10)	617.56 (310.44)	749.62 (473.77)
Distance to water (m, SD)	6695.73 (7745.45)	7762.31 (8096.52)	6364.54 (7074.80)
Age (years, SD)	29.35 (11.42)	28.17 (10.84)	30.39 (10.97)
Number of household members (*N*, SD)	6.57 (3.08)	6.67 (3.08)	6.78 (3.29)

For dichotomized risk factors, the weighted counts and weighted percentages for each category are provided. For continuous risk factors, the mean and standard deviation (SD) are provided.

*N* number of individuals, *mm* millimeters, *m* meters, *ITN* insecticide-treated net.

* Co-infections are included for both the *P. vivax* and *P. falciparum* marginal infection counts.

### Risk factors

In order to identify “who” was being infected by *P. vivax*, we performed a risk factor analysis that included common malaria exposures, or covariates. We also performed a risk factor analysis with *P. falciparum* as the outcome of interest for comparison. Risk factors were estimated using prevalence odds ratios (pORs) that were adjusted for confounding using inverse probability weights (IPWs). IPWs were calculated with a supervised machine learning approach: a spatially cross-validated super learner algorithm. However, the super learner candidate library was reduced to a regression model (i.e. linear regression or logistic regression for continuous versus categorical variables) in 9/11 models in favor of convergence or better IPW stability (Supplementary Table 6). For most covariates, IPWs were relatively stable, with some imbalances in housing materials and distance from healthcare facilities, and to a lesser extent, in education, farming, and wealth, potentially indicating lingering issues in structural positivity or residual confounding (Supplementary Fig. 6; Supplementary Table 5). Similarly, for most covariates, IPWs resulted in a considerable decrease in the average correlation among risk factors as compared to unadjusted baseline correlations (mean fold-reduction: 3.73, range: 1.10–7.37; Supplementary Fig. 7). However, for two risk factors: HIV status and precipitation, the maximum IPW-based correlation exceeded the maximum baseline correlation, which may indicate residual confounding.

**Fig. 2:**
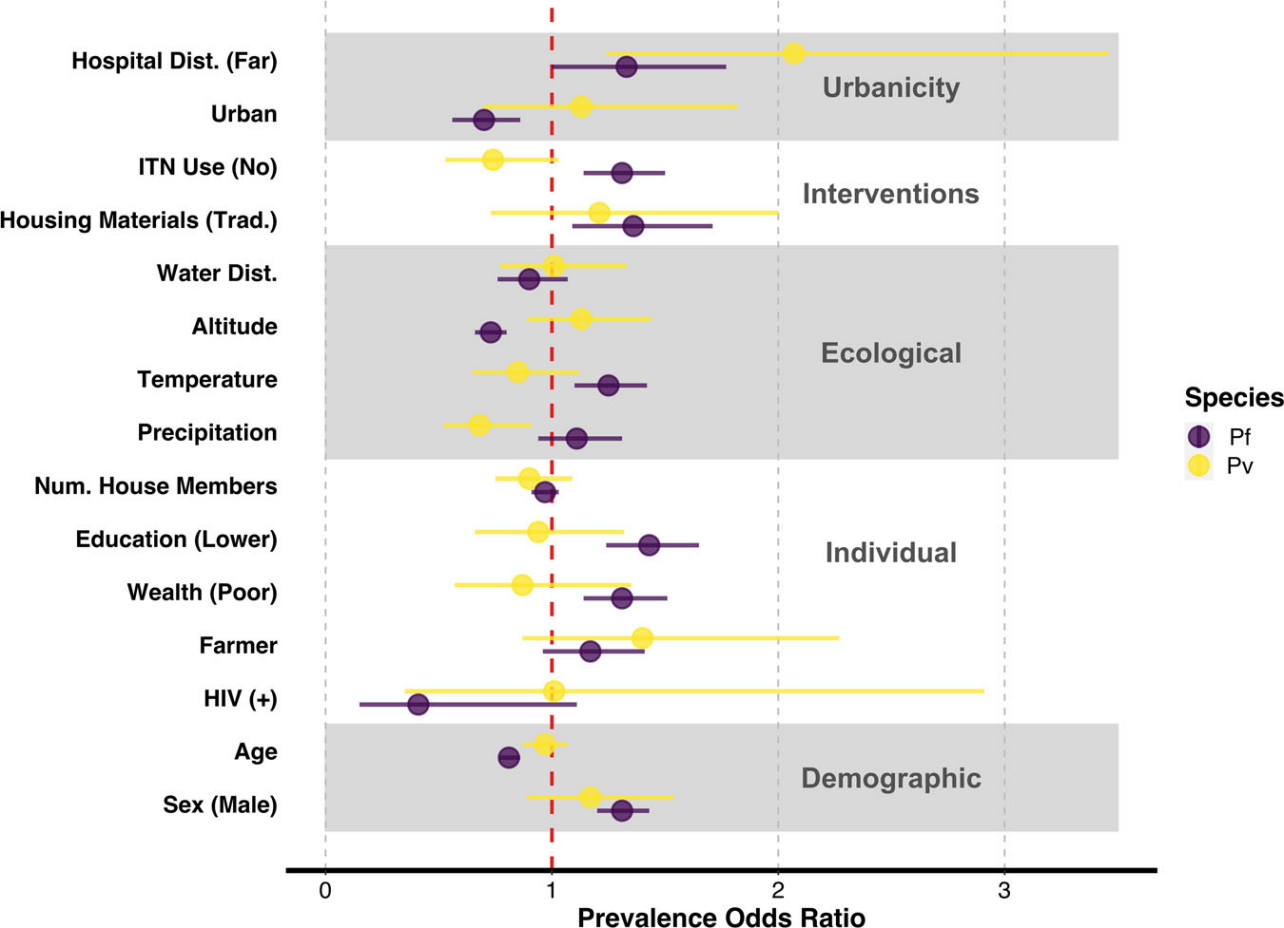
Inverse probability weight adjusted prevalence odds ratios for expected malaria risk factors. Risk factor point estimates and associated 95% confidence intervals are displayed for *P. vivax* and *P. falciparum*, respectively. Risk factors associated with *P. vivax* infection included precipitation and distance from healthcare facilities, while risk factors were associated with *P. falciparum* infection, included: urbanicity, housing materials, ITN use, altitude, temperature, education, wealth, age, and biological sex. The unadjusted pORs effect estimates and confidence intervals as well as the IPW-pORs are provided in Supplementary Table 7 for reference. Hospital Dist distance to healthcare facilities, Water Dist. distance to water, Trad. traditional, Num. House Members number of household members, ITN insecticide-treated net.

When *P. vivax* was considered as the outcome of interest, higher-levels of precipitation were associated with reduced prevalence (IPW-pOR: 0.68, 95% CI: 0.52, 0.91) while further distances from healthcare facilities were associated with increased prevalence (IPW-pOR: 2.07, 95% CI: 1.24, 3.46; Fig. 2). In conducting a sensitivity analysis on the coding of net-use, we found that our primary exposure, insecticide-treated net (ITN) use, contained the null for both the unweighted (Supplementary Table 7) and IPW-approach (Fig. 2) but that the DHS long-lasting insecticide net use variable only contained the null for the unweighted approach (OR: 0.75, 95% CI: 0.55, 1.01). For the IPW-approach, lack of long-lasting insecticide net use was associated with increased *P. vivax* prevalence (IPW-pOR: 0.70, 95% CI: 0.52, 0.96).

In contrast, when considering *P. falciparum* infections as the outcome of interest, several risk factors were associated with increased prevalence: lack of ITN use (IPW-pOR: 1.31, 95% CI: 1.14, 1.50), temperature (IPW-pOR: 1.25, 95% CI: 1.10, 1.42), lower levels of education (IPW-pOR: 1.43, 95% CI: 1.24, 1.65), low levels of wealth (PW-pOR: 1.31, 95% CI: 1.14, 1.51), traditional housing materials (PW-pOR: 1.36, 95% CI: 1.09, 1.71), and being male (IPW-pOR: 1.31, 95% CI: 1.20, 1.43). Additionally, three risk factors were associated with decreased prevalence: an urban setting (IPW-pOR: 0.70, 95% CI: 0.56, 0.86), increasing altitude (IPW-pOR: 0.73, 95% CI: 0.66, 0.80), and older age (IPW-pOR: 0.81, 95% CI: 0.77, 0.86; Fig. 2).

Based on our *post hoc* power calculations for *P. vivax*, we were able to detect approximate pOR estimates of at least 1.55, 1.36, 1.31 with at least 80% power when the exposure probability was 10%, 25%, and 50%, respectively. In contrast, for *P. falciparum*, we were able to detect approximate pOR estimates of at least 1.18, 1.12, 1.10 with at least 80% power when the exposure probability was 10%, 25%, and 50%, respectively (Supplementary Fig. 10).

**Fig. 3:**
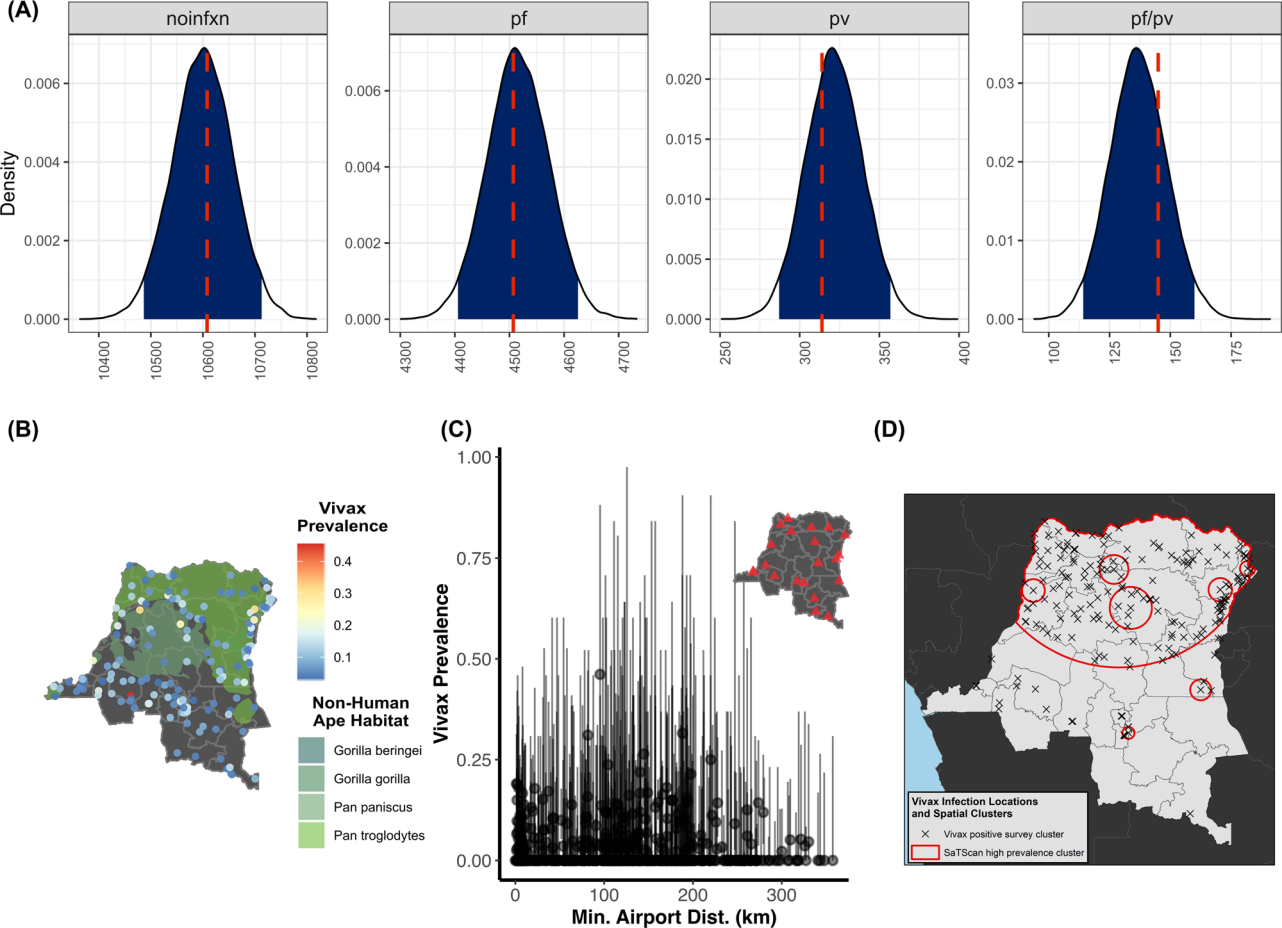
Non-parametric risk factor analysis and spatial clustering of *P. vivax* Infections. **A** Composition of *P. vivax* and *P. falciparum* co-infections. The expected versus observed composition of *P. vivax* and *P. falciparum* infections were explored using a multinomial probability likelihood model testing for independent acquisition of each species. To incorporate DHS sample weights, infections were rounded to the nearest integer. The plot shows the expected distribution for individuals without infection (“noinfxn”), *P. falciparum* infections (“pf”), *P. vivax* infections (“pv”), and *P. falciparum*–*P. vivax* co-infections (“pf/pv”). The blue shading indicates the 95% bootstrapped interval and the red-dotted line indicates the observed number of cases for each infection category. Overall, the observed distribution of each infection composition was explained well by each species occurring independently, which suggests that there were no interspecies infection synergism or antagonism. **B** Overlap with non-human ape habitats and positive *P. vivax* clusters. Overall, *P. vivax* prevalence did not appear to be associated with non-human ape habitat distribution (prevalence displayed as a proportion). This lack of a *P. vivax*-non-human ape association was recapitulated with permutation testing. Clusters with *P. vivax* infections are shaded on a blue–red spectrum with respect to the cluster-level prevalence, with the distribution of each non-human ape habitat is indicated in shades of green. **C** Correlation of *P. vivax* cluster prevalence with airport distance. The point estimate and 95% confidence interval for each cluster is shown as a point-range. Standard errors for the confidence intervals were calculated with the binomial exact method: to incorporate DHS weights, we rounded to the nearest integer. Airport distance was used as a proxy for the potentiality of *P. vivax* flight importation risk, or “airport malaria” risk. Overall, there did not appear to be a correlation between this variable and *P. vivax* prevalence (energy-correlation: 0.07). **D** Cluster detection of *P. vivax* infections. *P. vivax* infections appeared to be clustered in the northern and northeastern regions of the DRC with a small cluster also indicated in the center of the country.

A subset of risk factors were evaluated with non-parametric approaches due to concerns of traditional risk-factor model assumption violations or data limitations. These risk factors included: the Duffy-negative phenotype, *P. falciparum*–*P. vivax* infection interference, overlap with non-human ape habitats, and proximities to airports, as a proxy for importation of *P. vivax* via airline travel. Among those individuals infected with *P. vivax* and included in our study, three hosts were genotyped as heterozygous (−33T:T/C) at the loci associated with Duffy-negative phenotype (Prevalence: 0.64%, 95% CI: 0.13, 1.87%). Given that only *P. vivax* infected individuals were genotyped at the Duffy antigen loci, the overall prevalence of putative Duffy-positive phenotype was not generalizable to the entire DRC. From our cross-species interference model that assumes independent acquisition of infections from a multinomial likelihood, we failed to find any evidence of interactions between *P. falciparum*–*P. vivax* co-infections (Fig. 3A). This result suggests that infection with *P. falciparum* does not inhibit or promote *P. vivax* infection and vice-versa. Similarly, using permutation testing, we did not find an association between non-human ape habitats and *P. vivax* cluster prevalence (one-sided *p* > 0.05; Fig. 3B) nor a correlation between *P. vivax* prevalence and airport proximity (Fig. 3C).

### Spatial distribution of *P. vivax*

After identifying *P. vivax* risk factors, we wanted to identify “where” *P. vivax* infections were occurring. The spatial distribution of *P. vivax* was first assessed for disease clustering with ‘SaTScan‘ and using measures of autocorrelation with Moran’s I. We then modeled *P. vivax* prevalence across the DRC using Bayesian mixed spatial models that included risk factors that were previously identified as significant. Bayesian mixed spatial models were considered at two levels: (1) the province-level (areal model) and (2) the cluster-level (Gaussian spatial process model). Province-based spatial models are important for intervention-planning, as most interventions in the DRC are implemented at the province-level. However, cluster-level models with Gaussian processes may be more representative of the intrinsic malaria distribution under the assumption of a continuous spatial process.

A large, significant cluster of *P. vivax* was detected in the northern provinces of the DRC using ‘SaTScan‘ (Fig. 3D). Several smaller clusters were circumscript within the larger northern cluster and occurred within the north-central and north-eastern provinces. All clusters exhibited significantly elevated prevalence estimates relative to neighboring clusters with one-sided *p* values < 0.05.

**Fig. 4:**
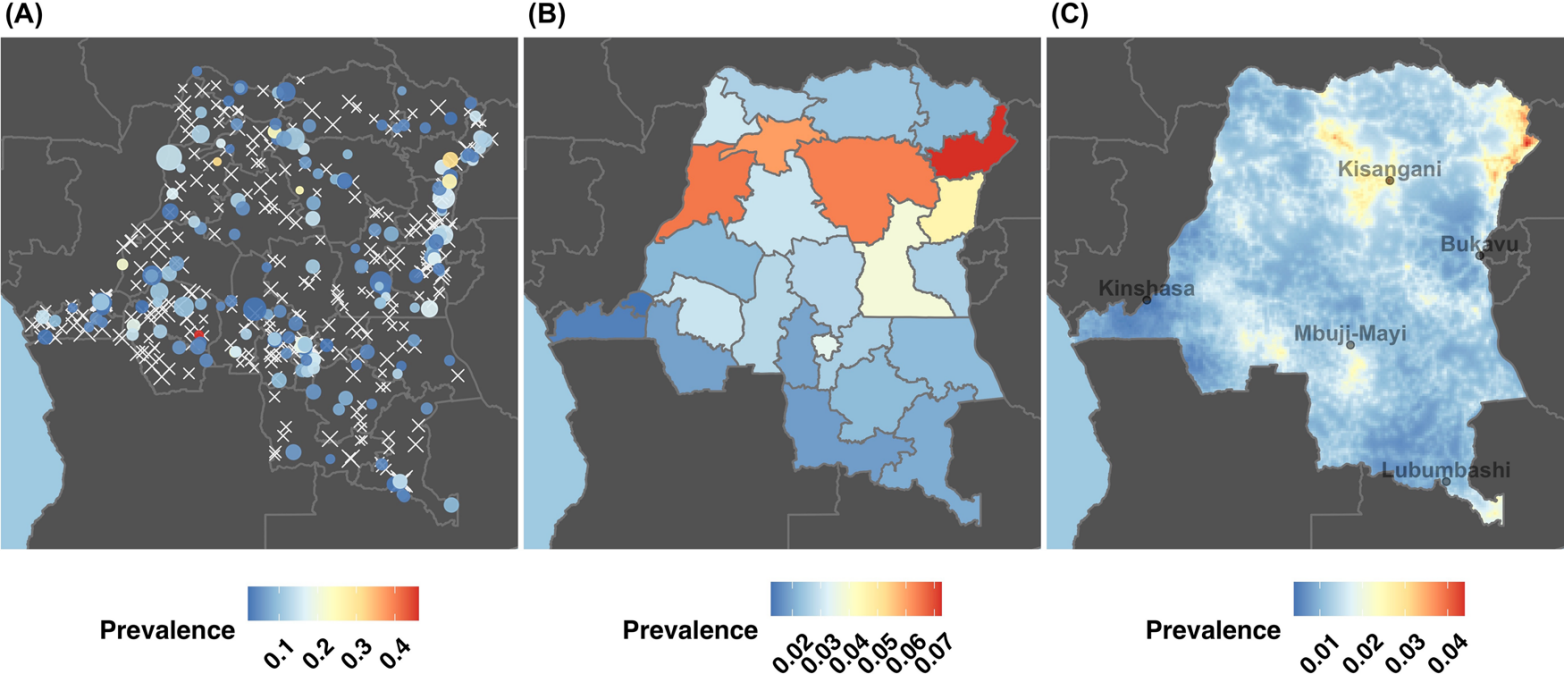
The distribution of *P. vivax* infections across the Democratic Republic of the Congo. For each map, the prevalence (as a proportion) is indicated along a blue–red spectrum (prevalence ranges and color scales differ between **A**–**C**). **A** Each cluster that contained a *P. vivax* infection is indicated along the blue–red prevalence spectrum, while clusters that lacked *P. vivax* infections are indicated with white X-marks. The scale of the cluster-mark reflects the cluster sample size (weighted-range: 1.41–269.63). *P. vivax* infections appeared to be diffusely spread throughout the country with cluster prevalences ranging from 0 to 46.15%. The cluster with the greatest prevalence, located in the Southeast near the Angola border, contained 6/13 infections. However, when DHS sampling weights were considered, this cluster was shrunk considerably. In addition, several surrounding clusters had no infections, resulting in less evidence for a focal region of high prevalence on spatial modeling: as such, its point-prevalence should not be overinterpreted. **B** The posterior mean prevalence estimates for the province-level model, which show that *P. vivax* infections appeared to be slightly more common in the north and northeast. **C** The posterior mean prevalence estimates that were interpolated across the DRC from the cluster-level model indicated a diffuse, low-level of *P. vivax* prevalence across the country with a few regions of higher prevalence. These focal regions of higher prevalence appeared to be concentrated in the northern and northeastern regions and to a lesser degree in the South. However, when considering the cluster-level model prevalences on a broader scale, ~99% of predicted prevalences are below the national average, which suggests a diffuse pattern of low prevalence across the DRC.

When considering spatial autocorrelation, we found that the province-level showed a slight signal of structure for *P. vivax* prevalence (Moran’s I: 0.16; one-sided *p* = 0.05), but this structure did not hold at the cluster-level (Moran’s I: 0.02; one-sided *p* > 0.05). Among the *P. vivax* province-level models considered, means-fitted province prevalences ranged from 1.25 to 7.26% (Fig. 4A). Standard errors for the province prevalence estimates ranged from 0.24 to 1.16% (Supplementary Fig. 9B).

When we modeled the spatial distribution of *P. vivax* at the cluster-level, *P. vivax* predicted prevalence ranged from 0.39 to 4.40% across the DRC (Fig. 4B). The standard errors around the prevalence predictions ranged from 0.26 to 4.18% (Supplementary Fig. 9C). *P. vivax* prevalence predictions were less than the observed national prevalence in ~99% of predicted cell locations.

Spatial model parameter posteriors and effective sampling sizes for both the province-level and cluster-level model are available in Supplementary Table 8. Similarly, the raster surfaces for the precipitation and distance from healthcare facilities covariates are displayed in Supplementary Fig. 8.

### *P. vivax* mitochondrial genetic distances

To identify the phylogeographic affinities of DRC *P. vivax*, we performed a simple genetic analysis comparing the mitochondrial genome (mtDNA) of our three DRC *P. vivax* samples with publicly available isolates. Among the three sequenced DRC *P. vivax* samples that underwent next generation sequencing with hybrid selection enrichment, we achieved high-quality coverage in ≥98.0% of the mtDNA (5× coverage with MQ ≥ 10, BQ ≥ 20). Among the 655 out of 705 publicly available Illumina sequenced *P. vivax* globally sourced and DRC isolates that passed QC-thresholds, we identified 97 biallelic single nucleotide polymorphisms (SNPs) and 102 unique mitochondrial haplotypes. When removing conserved haplotypes within countries, we identified 142 country-unique mitochondrial haplotypes (*N.B*. some identical haplotypes are shared between countries; Supplementary Fig. 11). Among the global contemporary isolates, the *P. vivax* populations from China and Cambodia showed the greatest within-population mtDNA nucleotide diversity while within-population haplotype diversity appeared to be greatest in the population from Vietnam. Overall, there was limited within-population nucleotide and haplotype diversity among the isolates from the DRC (Supplementary Table 9).

**Fig. 5:**
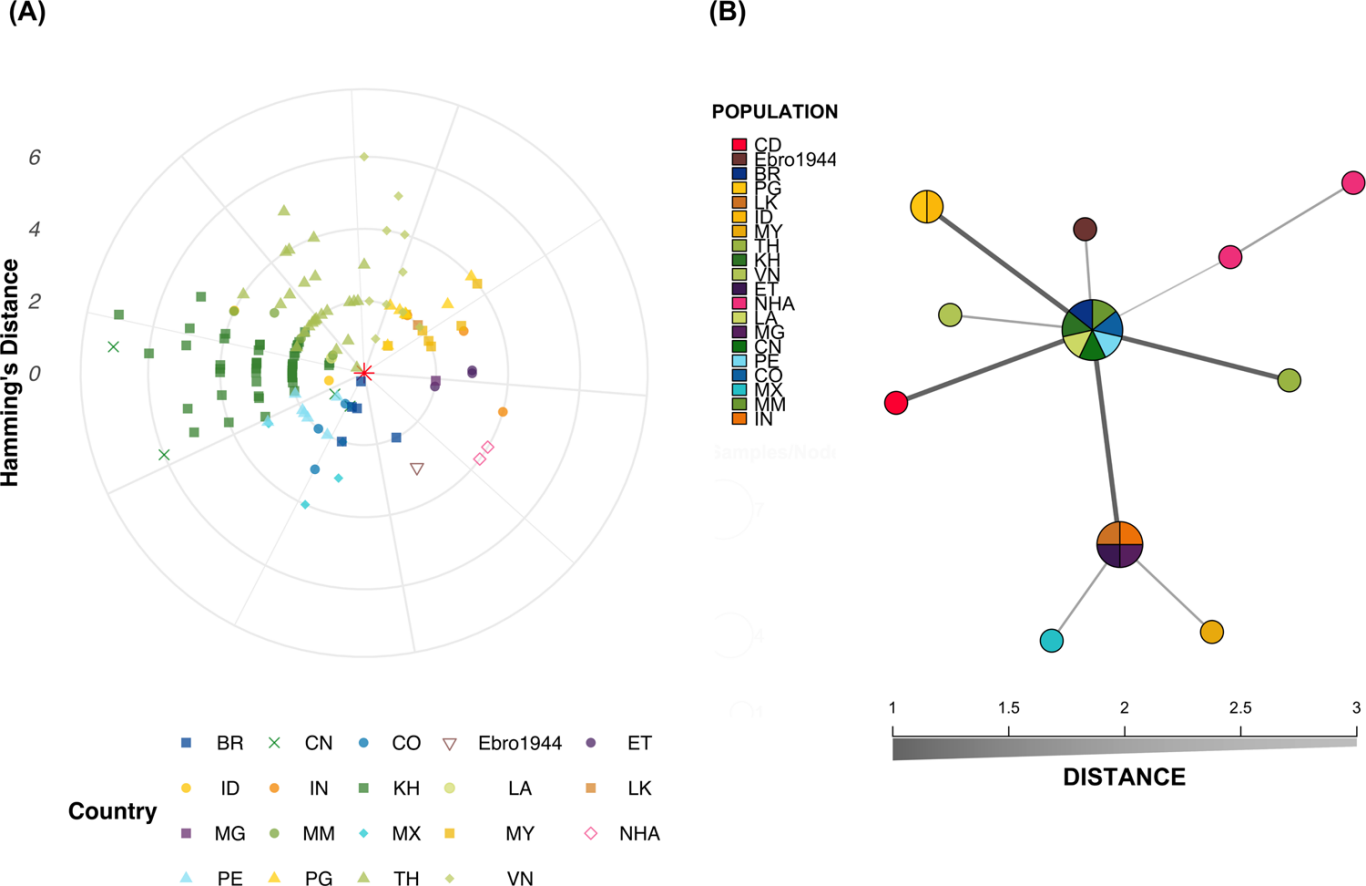
*P. vivax* mitochondrial Hamming’s distances and minimum-spanning network. Comparison of DRC *P. vivax* with over 700 globally sourced *P. vivax* isolates yielded 142 within-country unique consensus haplotypes and 102 overall unique consensus haplotypes, as duplicates existed within countries. In the instance of the DRC consensus haplotypes (*n* = 2/3), one isolate was identical except for ten missing sites. As a result, the two DRC consensus haplotypes were reduced to a single sequence and used in the above figures. **A** The DRC consensus haplotype is depicted in the center (red asterisk) and orbited by the other 140 consensus haplotypes. Distances from the center are determined by the number of base-pair differences while the relative position along the orbit is arbitrary and clustered only for visualization aid. Isolates from the Americas are colored in shades of blue and include Brazil (BR), Colombia (CO), Mexico (MX), and Peru (PE) Isolates from Asia are indicated in shades of yellow–green and include China (CN), Indonesia (ID), Cambodia (KH), Laos (LA), Myanmar (MM), Malaysia (MY), Papua New Guinea (PG), Thailand (TH), and Vietnam (VN). India (IN) and Sri Lanka (LK) are indicated in shades of orange, while Ethiopia (ET) and Madagascar (MG) are indicated in shades of purple. Finally, the Democratic Republic of the Congo (DRC) is shown in red and non-human apes (NHA) are shown in magenta. The historical sample from the Ebro Delta in Spain dating to 1944 (Ebro1944) is colored in brown. **B** A representative consensus haplotype from each country and both unique non-human ape sequences were selected as input into the minimum-spanning tree. Overall, there are similarities among the haplotypes from both the Americas and Asia, as well as the DRC consensus haplotype, although to a lesser degree. In terms of base-pair differences (Hamming’s distance), the DRC mitochondrial consensus haplotype differed by three and four bases to the Ebro1944 and the non-human ape consensus haplotypes, respectively. All unique consensus haplotypes with respect to country of origin are provided for comparison and context (Supplementary Fig. 11).

Direct visualization of the mitochondrial consensus haplotypes indicated that the DRC consensus haplotype was identical to a consensus haplotype from Brazil (*n* = 1 isolate) and Thailand (*n* = 3 isolates), as well as the Sal1 *P. vivax* lab strain consensus haplotype (base-pair difference range: 0–7). In contrast, the DRC mitochondrial consensus haplotype differed from Ebro-1944 and the non-human ape mitochondrial consensus haplotypes by three and four bases, respectively (Fig. 5A; Supplementary Fig. 11).

Considering Hamming’s genetic distances, we found that the DRC samples shared a low level of relatedness with contemporary isolates from multiple regions, including both Asia and South America (Fig. 5A). When considering a minimum-spanning network on a representative consensus haplotype from each country, we found that the DRC samples appeared to be most closely associated with contemporary *P. vivax* strains (Fig. 5B).

## Discussion

*P. vivax* infections among adults in the DRC are more common than previously realized. From our spatially robust dataset, we detected 467 *P. vivax* infections corresponding to a DRC national prevalence of 2.96% (95% CI_weighted_: 2.28, 3.65%). Among those infected, nearly all were Duffy-negative (464/467, 99.36%).

Risk-factors typically associated with *P. vivax* infection included precipitation and distance from healthcare facilities. The negative relationship between *P. vivax* prevalence and precipitation differed from other previous studies^10,11^, although the underlying effect is likely complicated by several ecological factors, such as temporal components, vector habitats, seasonality, altitude, and temperature^12^. Similarly, increased prevalence of falciparum malaria has previously been associated with access to healthcare resources in northern Ghana^13^ as well as other regions. These risk factors suggest that individuals that likely have less access to healthcare resources, particularly those who may be in climates that facilitate vivax malaria transmission, are more likely to be infected with *P. vivax*. However, the cluster-level spatial parameter estimate indicated that individuals further from healthcare facilities had lower associated *P. vivax* prevalences, possibly hinting at a complicated interaction between urbanicity and healthcare resources versus prevalence.

Overall, the *P. vivax* malaria risk factors differed from risk factors found for *P. falciparum* in this study using the same methodological approach. In addition, infection with *P. falciparum* did not seem to inhibit infection with *P. vivax* and vice-versa. This contrast between *P. vivax* and *P. falciparum* risk factors may indicate different processes of transmission, with vivax-specific factors including: a shortened intrinsic period which may cause decreased efficacy of typical antimalarial interventions, hypnozoite infections resulting in relapse infections despite individual uptake of antimalarial strategies or behaviors, or different vector capacities^14^. For example, net use was not associated with a protective effect in *P. vivax*, which may reflect a hypnozoite reservoir or long-term carriage of parasites. As a result, despite being sympatric infections, *P. falciparum* and *P. vivax* risk factors may diverge due to differences in transmission, and therefore, may not be expected to overlap.

*P. vivax* infections were found throughout the entire country with a few focal regions of relatively high prevalence. The highest prevalence and clustering of *P. vivax* infections was found in north and northeastern regions, particularly in the Ituri province. This may be due to cross-border migration with South Sudan and Uganda, which are near countries that are endemic for *P. vivax* (*P. vivax* infections have been reported in both countries)^5,7^. In 2013, the United Nations noted that these regions had a large concentration of internally displaced persons and refugees, which qualitatively adds to this hypothesis^15^. Future *P. vivax* epidemiological studies in the DRC should consider collecting human mobility data (e.g. cell-phone data), particularly with respect to Kinshasa and regions along the northeastern border where interactions with Duffy-positive immigrants may be more frequent. With human mobility data, it may be possible to capture putative transmission chains between Duffy-positive immigrants and DRC Duffy-negative inhabitants that would help to characterize the extent of secondary transmission and potential forward propagation of disease.

The sources of infection in other regions in the DRC was less clear, where most prevalence estimates ranged from ~0 to 2%. The scattering of infections across most of the DRC, as indicated by the maps and more than one-third of all *P. vivax* infected clusters containing only a single infection, suggests that *P. vivax* is diffusely distributed at a relatively low prevalence across most of the DRC. This essentially flat distribution of vivax malaria across the DRC contrasts the broad spatial distribution of *P. falciparum* infections previously observed in the 2007 and 2013 DRC DHS^16,17^. As a result, we suggest that *P. vivax* has been unable to gain a foothold in the region and is persisting rather than breaking out.

The relatively large differences in our DRC *P. vivax* and the non-human ape mitochondrial genomes negates recent zoonotic transmission as the source of DRC *P. vivax*^18^. This conclusion is strengthened by the lack of a significant relationship between *P. vivax* prevalence and non-human ape habitats. Similarly, we found that the now extinct European *P. vivax* strain, taken from a historical sample originating from the Ebro Delta in Spain, circa 1944^19,20^, differed by several bases from our DRC *P. vivax* consensus haplotypes. Instead, our DRC mitochondrial consensus haplotype was identical to consensus haplotypes found in Brazil, Thailand, and the Sal1 lab strain. This finding suggests that the DRC *P. vivax* strains fall within the mitochondrial diversity observed in contemporary *P. vivax* strains, although low levels of diversity and geographic structure precluded assessment using phylogenetic approaches. While the exact phylogeographic affiliations of these DRC *P. vivax* isolates is challenging to pinpoint with these mitochondrial genomes alone, we provide strong evidence that these infections did not originate from non-human apes and are likely distinct from the extinct European *P. vivax* circulating in the 1940s.

Although the mitochondrial genome is a non-recombining region with putatively neutral SNPs that is ideal for phylogenetic analysis, the few number of segregating sites precluded assessment of fine-scale geographic affiliations from the mtDNA. This relative lack of informative sites is consistent with previous reports^21^, but may also be an artifact of our small sample sizes in specific locations or our conservative approach to variant filtering. In addition, among the DRC mtDNA sequences, the variant at site 5910 was originally heterozygous and should be considered a low-confidence SNP. Future work with additional biological material to improve the likelihood of successful genomic sequencing will be needed to capture more genomic information allowing the application of more sophisticated methodological approaches such as coalescent modeling and spatio-demographic inference. Determining if DRC *P. vivax* has been recently imported, or is a distinct clade that has resulted from long-standing, neglected endemic transmission—as suggested in Mauritania^22^—will be informative for policymakers to determine the feasibility and urgency of vivax control and elimination efforts. In addition, with *P. vivax* whole genomes, signatures of selection for adaptation to the Duffy-negative host can be evaluated, which would help to better characterize and predict the threat of Duffy-negative transmission and disease.

The main limitations of our study are the cross-sectional design, which limits inference of effects with a temporal component (e.g. seasonality), the DHS sampling design, which restricts the study population largely to asymptomatic individuals and misses critical age groups^23^, the proxies and spatial resolution of the various risk factors, and the aforementioned small number of high-quality DRC sequences generated. In addition, the Φ-parameter and the σ^2^-parameter in the cluster-level spatial model, as well as the ρ-parameter in the province-level spatial model, remained somewhat unstable, potentially reflecting the complicated spatial autocorrelation among the *P. vivax* prevalences. Similarly, although limits of power were explored, it is possible that *P. vivax* specific risk factors were missed given the relatively few infections and wide confidence intervals. A spatially robust, large prospective cohort study across all ages and across multiple seasons could address many of these limitations, but costs are likely to be prohibitive.

Until recently, *P. vivax*, was an unrecognized cause of disease in SSA. The DRC is a critical region for the study of malaria in SSA due to its geographic size, central location, and the evidence that it bridges East and West Africa malaria^8,9^. Although previous studies have screened large populations in SSA for *P. vivax*^6,24–28^, this study provides a systematic and nationally representative survey of *P. vivax* in a SSA country not considered endemic for the disease. We demonstrated that *P. vivax* is circulating at prevalences higher than previously thought, despite a high frequency of Duffy-negativity^7^. However, *P. vivax* infections were associated with few risk factors, were spread diffusely throughout the country, and did not have a clear genetic ancestry based on the mitochondrial genome, with the exception that these are likely not zoonotic infections from non-human apes and likely not an ancestral remnant^18–21^. Instead, the DRC *P. vivax* strains appear to be contemporary, prompting three possible and non-mutually exclusive explanations: (1) these infections are frequently present at sub-microscopic or low parasitemia that potentially limit transmissibility^29^, (2) these infections are the result of continual importation of *P. vivax* with limited forward-propagation, or (3) infections are the result of long-standing relapse as primaquine is not routinely administered in SSA. Although there are numerous other explanations, all three of these explanations are consistent with previous work that suggests *P. vivax* infections among Duffy-negative individuals are frequently mild and asymptomatic compared with Duffy-positive individuals^5,6^. Finally, emerging research suggests that genotypically Duffy-negative hosts express the Duffy antigen among erythroid progenitors in the bone marrow and that *P. vivax* gametocytes are able to mature and proliferate in the bone marrow of non-human primate animal models^30,31^. Collectively, this suggests that *P. vivax* in SSA may be persisting as low parasitemic, asymptomatic infections, by hiding in the bone marrow or other tissues containing early progenitor red blood cells of Duffy-negative hosts^30–32^. As a result, if these infections are typically asymptomatic and present with lower parasitemia that limit transmissibility, they pose a limited morbidity cost to the individual and an overall low public health threat.

*P. vivax* infections among Duffy-negative individuals appear to be occurring throughout SSA^6,24–28^. However, the current distribution and low prevalence of vivax malaria in SSA supports continued investments targeting *P. falciparum* as likely having the greatest impact on malaria control, morbidity, and mortality. Future efforts targeted at the DRC malaria elimination end-game may need to consider vivax-specific interventions.

## Methods

### Study participants and malaria detection

We studied men and women aged 15–59 years and 15–49 years, respectively, that were surveyed in the 2013–2014 DRC DHS. Each participant answered an extensive questionnaire and provided a dried blood spot (DBS) for HIV and other biomarker screening. Spatial and ecological data were collected for each sampling cluster (Supplementary Materials: Covariate Feature Engineering, Spatial and Raster Feature Engineering). We extracted DNA from each DBS using Chelex-100 (Bio-Rad, Hercules, CA) and Saponin and then screened all participants for *P. vivax* using qPCR targeting the 18S ribosomal RNA gene^33^. Samples that screened positive by 18S-qPCR underwent reflex confirmatory screening using a nested-PCR assay targeting 18S rRNA (Supplementary Table 1)^34^. To ensure the quality of DNA extraction, we excluded samples that had previously failed to amplify human-beta-tubulin^17^ from analysis. Finally, participants were excluded if they had missing data or were not a part of the DHS sampling schematic (Fig. 1)^35^. This study reanalyzes previously published *P. falciparum* data that was generated with a *P. falciparum lactate* dehydrogenase gene qPCR approach (sample size differences are due to different inclusion criteria)^17^. This study was approved by the IRBs at the University of North Carolina at Chapel Hill and the Kinshasa School of Public Health.

### Duffy genotyping

All samples that initially screened positive underwent Host Duffy antigen/chemokine receptor (DARC) genotyping. The DARC genotype was determined using a previously validated High-Resolution Melt (HRM) assay (Supplementary Table 3)^36^. Genotypes that could not be definitely resolved by HRM were reconciled by Sanger sequencing^6^. In addition, HRM results were validated by sequencing ~10% samples (Supplementary Materials: Duffy-Genotyping).

### Risk factor modeling

*P.* *vivax* risk factors were identified from a comprehensive literature search and previous work from the 2013 to 2014 DRC DHS identifying *P. falciparum* risk factors^13,16,17,37^. Risk factors were derived from the DHS questionnaires and other open-data sources (Supplementary Materials: Covariate Feature Engineering) https://www.hotosm.org/^35,38–41^. All continuous risk factors were standardized in order to promote model stability and ease of comparability. For dichotomized risk factors, the a priori protective level was selected as the referent level (e.g. HIV-negative) or the largest group if a protective level was not obvious (e.g. female for biological sex).

For each risk factor, confounding covariates were identified using a directed acyclic diagram (DAG) built from our a priori causal framework of covariate and outcome relationships (Supplementary Fig. 3). To confirm manageable collinearity, we analyzed the energy correlation between all covariates (Supplementary Fig. 4). We then used IPW to obtain marginal structural models and account for confounding between our risk factors and outcome of interest, malaria^42–45^. IPWs were calculated with a super learner algorithm, which uses a loss-based approach with V-fold cross-validation to maximize predictions from an ensemble of candidate algorithms (Supplementary Table 4)^46^. We extended the standard super learner algorithm to account for spatial dependence among observations using spatial cross validation (Supplementary Materials: Inverse Probability Weights and Prevalence Odds Ratios; Supplementary Fig. 5)^47^. The super learner algorithm was originally selected for IPW calculations to account for known issues and biases of functional form in fitting the exposure dose-response curve^48^. However, as stated above, the super learner candidate library was reduced to a regression model in 9/11 models in favor of convergence or better IPW stability. IPWs and DHS sampling weights were accounted for under the assumption that the distribution of the sampling was independent of the distribution of confounding covariates, which allows for weights to be considered jointly, ${w}_{f}={w}_{s}\ast {w}_{{ipw}}$. We then used the ‘survey‘ R package to account for the DHS sampling design and to perform the generalized estimating equation (GEE) regression. The GEEs were calculated with a logit-link function and binomial variance to produce odds ratios. Finally, we performed power calculations given our study and sample characteristics to determine the extent of the power that we had to detect significant odds ratios (Supplementary Materials: post-hoc Power Calculations)

In addition, we considered several alternative *P. vivax* risk factors that could not be estimated with a parametric approach due to model assumption violations or a lack of data. These additional risk factors included: (1) the putative Duffy phenotype (Supplementary Materials: Duffy-Genotyping); (2) within-host interactions of *P. vivax* and *P. falciparum* using a multinomial likelihood-based model that assumes independent infection acquisition (Supplementary Materials: *P. falciparum*–*P. vivax* Co-infection Model)^49^; (3) interactions between non-human ape ranges and *P. vivax* cluster-level prevalences using permutation tests (Supplementary Materials: Overlap with Non-Human Ape Permutation Testing)^18,50^; and (4) the association between *P. vivax* cluster prevalence and the proximity to airports, as a proxy for importation of *P. vivax* via airline travel. Proximity to airports was calculated as the minimum greater-circle distance from each cluster to an airport that was classified as “medium” or “large” (Supplementary Materials: Covariate Feature Engineering).

### *P. vivax* spatial analyses and prevalence mapping

Spatial clustering of *P. vivax* was initially assessed using spatial scan statistics through the ‘SaTScan‘ (v9.6.1) platform^51^. A Poisson distribution of cases was assumed and the model was specified to detect only clusters of higher prevalence relative to neighboring survey cluster locations. A significance threshold of 0.05 was set for cluster detection.

We considered spatial autocorrelation with Moran’s I using a province adjacency matrix as well as a matrix of greater-circle distances between clusters^52^. Greater-circle distances were calculated using a geodesic approach^53^. Significance was evaluated using a permutation test with 100,000 iterations and a one-sided *p* value.

To determine the spatial distribution of *P. vivax*, we fit two types of Bayesian mixed spatial models: (1) a province-level areal model and (2) a cluster-level Gaussian spatial process model^54,55^. Both sets of spatial models were fit with generalized linear mixed models using the logistic link function and a binomial error distribution with a spatial random effect. The selected priors and the full model formulations are available in the Supplementary Materials: Bayesian Spatial Prevalence Models. For each of the respective spatial-levels, we used the identified significant risk factors in the model fitting. Spatial covariates were extracted from the Climate Hazards Group Infrared Precipitation with Stations (precipitation)^39^ using the ‘environmentalinformatics-marburg/heavyRain‘ wrapper and the Malaria Atlas Project (average walking travel times to health care facilities)^41^ to incorporate the significant risk factors that were identified (Supplementary Materials: Spatial and Raster Feature Engineering).

For simplicity, we assumed the WGS84 projection system throughout this analysis, which includes all risk factors with a spatial component, spatial covariates, and spatial models. This assumption is relatively minor as the DRC straddles the equator.

### *P. vivax* mitochondrial genomics

DNA quantity and quality was limited from the adult participants considered in this study taken from the 2013 to 2014 DRC DHS. However, DNA from children collected as part of the 2013–2014 DRC DHS was available at a higher quality and was able to be sequenced^56^. From this previous study, DNA from three previously identified samples was successfully amplified using the Illustra Genomic Phi V2 DNA Amplification Kit (GE Healthcare Life Sciences, Pittsburgh, PA) and sequencing libraries prepared with NEBNext Ultra DNA Library Prep Kit for Illumina (New England BioLabs Inc., Ipswich, MA). Amplified libraries were then enriched using a custom in-solution product (MYbaits) targeting the *P. vivax* genome (version 3.0; MYcroarray: The Oligo Library Company, Ann Arbor, MI). Enriched genomes were sequenced on MiSeq 150 base-pair paired-end and HiSeq2500 125 base-pair paired-end chemistry (Illumina, San Diego, CA) platforms. All subsequent analyses were limited to the mtDNA due to low coverage in the nuclear genome. Nucleotide variants were identified among all samples and unique consensus haplotypes were determined (Supplementary Materials: Variant Filtering and Consensus Haplotypes). Using these three DRC samples and the globally sourced mitochondrial alignments, we calculated basic population genetic statistic summaries, including: within-population nucleotide diversity, counts of unique consensus haplotypes, and between-sample Hamming’s distance (i.e. base-pair differences). For our basic genetic distance analysis, we removed duplicate consensus haplotypes within countries and visualized base-pair differences (Supplementary Materials: Population Genetic Statistics and Distances). Next, we subset the data to a representative consensus haplotype for each country and created a minimum-spanning network (Supplementary Materials: Population Genetic Statistics and Distances).

### Reporting summary

Further information on research design is available in the Nature Research Reporting Summary linked to this article.

## Supplementary information

Supplementary Information

Reporting Summary

## Data Availability

Due to data privacy considerations relating to the Demographic Health Survey, the molecular PCR results generated and analyzed during the current study are available from the corresponding author upon reasonable request. In addition, all epidemiological covariate data is available upon reasonable to the corresponding author, including intermediary files. The genomic data produced by the current study is available at the short reads archive bioproject: PRJNA725254.

## Code availability

All scripts and code used to generate these analyses are publicly available on Github (*Epidemiological Analyses*: github.com/nickbrazeau/VivID_Epi; *Population Genetic Analyses*: github.com/nickbrazeau/VivID_Seq). The custom scripts used in this analysis are available under the Github repositories: github.com/nickbrazeau/mlrwrapSL, github.com/IDEELResearch/vcfRmanip, github.com/nickbrazeau/icer.
