# Supplementary material for: The epidemiology of Plasmodium vivax among adults in the Democratic Republic of the Congo
Source: Nat Commun. 2021 Jul 7;12:4169. doi: 10.1038/s41467-021-24216-3 (PMC8263614; doi:10.1038/s41467-021-24216-3)
Supplement: Supplementary file 1 — Supplementary Information [file 41467_2021_24216_MOESM1_ESM.pdf]

Supplementary Information

Supplementary Note 1: Molecular Diagnostics 1
P. vivax Infection Detection 1
P. vivax Infection Assay Validation and Challenge 2
Duffy-Genotyping 4
Supplementary Note 2: Epidemiological Analyses 6
Study Population and Data Sources 6
Covariate Feature Engineering 7
P. falciparum-P. vivax Co-infection Model 11
Overlap with Non-Human Ape Permutation Testing 12
Inverse Probability Weights and Prevalence Odds Ratios 12
Spatial and Raster Feature Engineering 21
Bayesian Spatial Prevalence Models 21
post-hoc Power Calculations 26
Supplementary Note 3: Population Genetics 27
Publicly Available Whole Genome Sequences 27
Alignment, Quality Control, and Variant Discovery 27
Variant Filtering and Consensus Haplotypes 27
Population Genetic Statistics and Distances 29
Supplementary References 30

Supplementary Note 1: Molecular Diagnostics

P. vivax Infection Detection

DNA was extracted from dried blood spots using Chelex-100 (Bio-Rad, Hercules, CA) and Saponin as previously described.[1,2] *P. vivax* infections were detected using a two-stage approach that combined a TaqMan quantitative PCR (qPCR) assay targeting the 18S rRNA gene and a confirmatory nested-PCR assay.[3,4] A two-step approach was utilized to increase specificity and limit potential false positives given the range of cycle-threshold (CT) values considered.

For the qPCR assay, individual reactions were performed at a final volume of 18 µL: 5 µL of template DNA, 9 µL of FastStart Universal Probe Master Mix (ROX) (Roche Diagnostics, Indianapolis, IN), 0.36 µL of each primer at 20 µM, 0.36 µL of probe at 10 µM, and 2.92 µL of molecular grade water (**Supplementary Table 1**). All qPCR reactions were performed on a QuantStudio 6 Flex Real-Time PCR System (ThermoFisher Scientific, Waltham, MA, USA) using the following thermal cycling conditions: hold at 50°C for 2 minutes, denaturation at 95°C for 10 minutes, and 45 cycles of 95°C for 15 seconds and annealing at 60°C for 1 minute. All bulk qPCR reactions included two replicates of positive controls: 10-fold standard dilutions of a *P. vivax* 18S PCR plasmid clone (MRA-178, BEI Resources, Manassas, VA, USA) from 4,550 parasites/µL (10^{-4} ng/µL) to 4.55 parasites/µL (10^{-7} ng/µL), assuming 6 copies of 18S per parasite.[5,6] In addition, four negative template controls were added to each qPCR plate. For all qPCR runs, a threshold of 0.04 log change in Rn was set and results were then exported for analysis. Any samples with uncharacteristic amplification profiles, or samples with amplification prior to 13 cycles, were marked as undetermined.

To confirm the presence of *P. vivax* infections, all samples that were positive by qPCR underwent reflex confirmatory screening using a nested PCR assay.[4] The first round of the nested PCR assay targeted a 1.6-1.7 kilobase region of the 18S gene specific to the *Plasmodium* genus using the Plu1 and Plu5 primers (**Supplementary Table 1**). For the first round PCR, reactions were performed in a final volume of 25 µL: 5uL of sample DNA, 12.5uL HotStarTaq Mastermix (Qiagen, Venlo, Netherlands), 0.5 µL of 20 µM each of the forward and reverse primers, and 6.5 µL molecular grade water. PCR was performed on a BioRad T100 Thermal Cycler (Applied Biosystems, Foster City, CA, USA) using the following thermocycler conditions: 95°C for 15 minutes followed by 35 cycles of 94°C for 1 minute, annealing at 50°C for 1 minute, and extension at 72°C for 1 minute, with a final extension at 72°C for 10 minutes. Product from the first round of PCR was handled in a designated post-PCR section of the laboratory to inhibit contamination of pre-PCR work surfaces. The second round PCR reaction targeted a 121 base pair region specific to *P. vivax* using the rViv1 and rViv2 primers (**Supplementary Table 1**). The second round reaction recipe was identical to the first round reaction recipe with the exception that the PCR product from the first round was used as the DNA template for the second round. Thermal cycling conditions were the same with the exception of a raised annealing temperature of 62°C. Final PCR product was visualized using 5% ethidium bromide on a 1% agarose gel run in 1x Tris-borate-EDTA buffer at 100 volts for 1 hour. Positive confirmation of *P. vivax* was based on visualization of the 121 base pair PCR amplicon and was evaluated by two reviewers independently. A confirmed infection was only considered when reviewers were in agreement. Among the 579/17,972 qPCR-positive samples, the inter-observer agreement of the absence/presence of a PCR band was high (Agreement: 565/579, Cohen's $\kappa = 0.80$, $p < 0.05$).

Assay	Primer	Sequence	Ref.
P. vivax Diagnostic qPCR	PvForward	5'-ACGCTTCTAGATTAATCCACATAACT	3
	PvReverse	5'-ATTTACTCAAAGTAACAAGGACTTCCAAGC	
	Pv-probe (FAM-IowaBlack)	5'-TTCGTATCG/ZEN/ACTTTGTGCGCATTTTGC	
P. vivax Confirmatory PCR	Plu1	5'-TCAAAGATTAAGCCATGCAAGTGA	4
	Plu5	5'- CCTGTTGTTGCCTTAAACTCC	
	rVivi1	5'-CGCTTCTAGCTTAATCCACATAACTGATAC	
	rVivi2	5'-ACTTCCAAGCCGAAGCAAAGAAAGTCCTTA	

Supplementary Table 1 - *P. vivax* Infection Detection Assays: The adapted protocols used for diagnostic qPCR and confirmatory PCR for the detection of *P. vivax* infections. For the qPCR assay, the probe differs from Srisutham *et al.* 2017 in the use of FAM as the fluorescent label, an additional ZEN quencher, and the use of Iowa Black as the 3' terminus quencher. All probes and primers were synthesized by Integrated Device Technology, Inc. (San Jose, CA, USA).

P. vivax Infection Assay Validation and Challenge

To assess the lower limit of detection of the *P. vivax* TaqMan assay, the *P. vivax* 18S qPCR plasmid clone (MRA-178, BEI Resources, Manassas, VA, USA) was diluted to $1x10^{-6}$ ng/µL and then serially diluted 2-fold to a lower limit of $0.03125x10^{-6}$ ng/µL. The assay was then run with 12 replicates for the $1x10^{-6}$ and five $1x10^{-5}$ dilutions and with 22 replicates for the remaining dilutions (**Supplementary Table 2**). Cycle threshold values were read at a log change in Rn value of 0.04—identical to settings used for the detection of *P. vivax* infections. Overall, the 95% lower limit of detection for *P. vivax* was approximately 7 parasites/µL (assuming 6 copies of 18S per parasite), or a DNA concentration of $1.5\ x10^{-7}$ ng/µL (**Supplementary Table 2**).

In addition, the specificity of the assay was challenged using 20 replicates of 18S plasmid concentrated at $1x10^{-4}$ ng/µL for *P. falciparum* (MRA-177, BEI Resources), *Plasmodium malariae* (MRA-179, BEI Resources), and *Plasmodium ovale* (MRA-180, BEI Resources), respectively.[5,6] In all challenges, no off-target amplification was observed (**Supplementary Figure 1**).

Target concentration ($x10^{-6}$) ng/µL	No. Tested	No. Detected	Percent Detected	Average parasites/µL	Mean Ct (of detected)
Pv 18S 1	12	12	100	45.5	32.680
Pv 18S 0.5	12	12	100	22.75	34.398
Pv 18S 0.25	22	22	100	11.375	35.978
Pv 18S 0.125	22	20	90.91	5.6875	37.448
Pv 18S 0.0625	22	14	63.64	2.84375	38.691
Pv 18S 0.03125	22	6	27.27	1.421875	38.538
Pf 18S 100	20	0	0	--	--
Pm 18S 100	20	0	0	--	--
Po 18S 100	20	0	0	--	--

Supplementary Table 2 - *P. vivax* Lower Limit of Detection and Off-Target Amplification: The concentrations of the 18S plasmid for each of the *Plasmomdium spp.: P. vivax* (Pv), *P. falciparum* (Pf), *P. malariae* (Pm), and *P. ovale* (Po), are provided alongside the number of replicates tested. The percent detected was calculated as the proportion of replicates identified with a cycle threshold value less than 45 among those tested. Among those samples that were detected, the mean cycle threshold value is provided. *Abbreviations*: No. – number, Ct – cycle threshold.

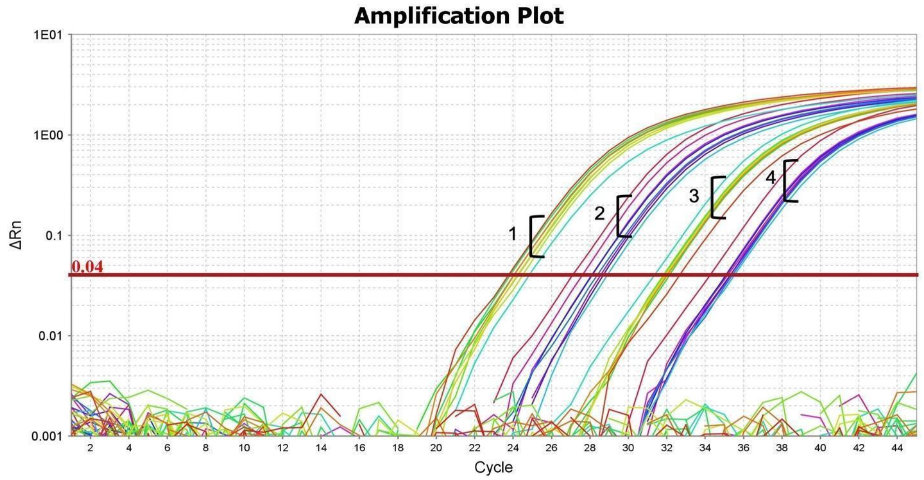

Supplementary Figure 1 - *P. vivax* qPCR Specificity Challenge: Amplification of *P. vivax* 18S plasmid dilutions containing 4,550 (1), 455 (2), 45.5 (3), and 4.55 (4) parasites/µL. Each *P. vivax* dilution was tested with eight replicates. Amplification failed for all 20 replicates of *P. falciparum*, *P. malariae,* and *P. ovale* 18S plasmids (concentration: $1x10^{-4}$ ng/µL).

Duffy-Genotyping

For each sample that was positive by qPCR, we used a previously validated high-resolution melt (HRM) assay to genotype the GATA-1 transcription factor (-33 T:C) point mutation that has been previously shown to silence Duffy Antigen/Chemokine Receptor (DARC) expression.[7,8] Each HRM reaction contained a final concentration of 1x MeltDoctor HRM Master Mix (Applied Biosystems, Foster City, CA, USA), 0.3 µM forward primer (DARCf), 0.3 µM reverse primer (DARCr), 100 pg of template DNA in a final volume of 20 µM (**Supplementary Table 3**). Reactions were performed using the following thermocycler conditions: denaturation at 95°C for 10 minutes, followed by 45 cycles of 95°C for 15 seconds, 60°C for 1 minute, 95°C for 10 seconds, 60°C for 1 minute, 95°C for 15 seconds, and 60°C for 15 seconds on a QuantStudio 6 Flex Real-Time PCR System (ThermoFisher Scientific, Waltham, MA, USA). Each HRM plate contained a DARC-positive (-33 C:C), DARC-negative (-33 T:C), and a non-template control which were used to call HRM results on each plate independently.

Samples that could not be definitively determined by HRM and a 10% random subset of *P. vivax* qPCR-positive samples underwent confirmatory Sanger sequencing genotyping at Eton Bioscience (Research Triangle, NC). PCR products were generated from a previously validated assay.[9] Final reactions contained 0.25 µL of FastStart High Fidelity Taq (Enzyme Blend; Roche©, Indianapolis, IN), 2.5 µL of 10x FastStart High Fidelity reaction buffer with 18 mM $MgCl_2$, 0.36 µM forward primer, 0.36 µM reverse primer, 250 µM dNTPs and 3 µL of template DNA in a volume of 25 µL. Reactions were amplified using the following thermocycler conditions: denaturation at 94°C for 15 minutes followed by 40 cycles of 94°C for 30 seconds, annealing at 58°C for 30 seconds, extension at 72°C for 90 seconds, and a final extension at 72°C for 10 minute on a BioRad T100 Thermal Cycler (Applied Biosystems®, Foster City, CA, USA). PCR products and Sanger sequences were also generated for a DARC-positive control (-33 C:C) and DARC-negative control (-33 T:C).

For each sample, forward and reverse sequences were analyzed using Geneious 10.1.3 (Biomatters Limited, Auckland, New Zealand). First, the 5' and 3' ends of each sequence were trimmed using Geneious `Trim Ends` tool with a 0.05 error probability limit. For each sample, forward and reverse sequences were then *de novo* assembled using the Geneious `Assembler` tool with the sensitivity flag set to "Highest Sensitivity/Slow". Of the 51 random samples sequenced, one sample was unable to be assembled due to low sequencing quality. Of the 17 samples that underwent confirmatory sequencing, all samples were assembled. The mapped sequences were then visually assessed for the DARC (-33 T:C) point mutation. Duffy-Genotypes by Sanger sequencing were concordant with the HRM-qPCR results among the remaining 50/51 samples selected for validation.

Assay	Primer	Sequence	Ref.
HRM Genotyping	DARCf	5’-CGTGGGGTAAGGCTTCCTGA	7
	DARCr	5’-CTGTGCAGACAGTTCCCCAT	
Confirmatory PCR	ESf	5’-GTGGGGTAAGGCTTCCTGAT	9
	ESr	5’-CAAACAGCAGGGGAAATGAG	

Supplementary Table 3 - DARC-Genotyping Primers: All samples that were positive by qPCR underwent genotyping at the Duffy Antigen/Chemokine Receptor (DARC) promoter region using High Resolution Melt (HRM) Analysis. A subset of randomly selected samples and those samples that could not be absolutely confirmed by HRM underwent confirmatory Sanger sequencing of a GATA-1 transcription factor amplicon that contained the region of interest.

Supplementary Note 2: Epidemiological Analyses

Study Population and Data Sources

In the Democratic Republic of the Congo (DRC), the Demographic Health Survey (DHS) aimed to create a nationally representative survey using a two-stage stratified cluster sampling design.[10] In the first stage, clusters, or enumeration area, were selected with a known and fixed probability. During the second stage, within each cluster, a subset of households were selected. Finally, among those adults residing in selected households, a subset were consented for HIV and other biomarker testing. To control for this sampling scheme, the DHS weights each individual with an inverse probability weight of selection, hereafter, sampling weight.[10]

The DRC 2013-2014 DHS-VI survey was conducted from August 2013 - September 2013 and November 2013 - February 2014. Specifically, DHS surveyors screened Kinshasa and surrounding areas from August 2013 - September 2013 and then subsequently administered the survey across the rest of the country from November 2013 - February 2014.

For each household, DHS surveyors acquired informed consent and administered a substantial questionnaire to all individuals that had slept in the household the night prior to the interview.[10] Individuals that permanently reside in the household are classified as *de jure* while individuals that were coincidentally in the household the night preceding the interview were classified as *de facto*.[10] Given that household variables were considered as potential malaria risk factors, we limited observations to the *de jure* population, as *de facto* individuals' homes may differ substantially from the home that they were visiting.[10]

Among those adults that agreed to undergo HIV and other biomarker testing, a dried blood spot (DBS) was taken. DBS were then punched into 96-well plates and associated barcodes were manually recorded in a spreadsheet in the DRC. The 96-well plates were then sent to the University of North Carolina-Chapel Hill (UNC) for malaria testing.

In total, 17,959/17,972 samples with properly formatted barcodes were screened by qPCR at UNC. These samples were then linked to the DHS HIV (AR) recode excluding the 288 samples that were contaminated during shipment from the DRC to UNC (one barcode was duplicated and excluded). On the initial merge, 17,859/17,959 samples were successfully linked. In order to recover more samples, we allowed for a one-character mismatch between the manually recorded DBS barcode and the DHS barcode among those samples that did not have a match in the preliminary merge. Using this strategy, we successfully recovered an additional 75 samples accounting for our total of 17,934/17,959 samples that were screened by qPCR. Among these 17,934 samples, 169 previously identified samples failed to amplify human beta-tubulin, which was used as a within-sample positive control, and thus, were excluded from the study population.[1] Of these 17,765 samples, 1,402 were missing geospatial data (44 clusters), 237 individuals were not *de jure* household members, and 535 have sampling-weights set to zero. We assumed that all samples lost due to shipping contamination, failure to amplify human beta-tubulin, barcode typos, and missing geospatial data were due to "accidents" and were missing completely at random or were excluded under the DHS sampling framework (i.e. missing sampling weights, *de jure*).[10] From this refined dataset, we had only 17/15,591 (0.11%) observations that were missing conditional on covariates considered in our study. As a result, after excluding those individuals with missing covariate observations, our final study sample size included 15,574 adults across 489 clusters (**Figure 1**).

Overall, we detected 467 *P. vivax* infections prior to applying DHS sampling weights. The DRC national weighted prevalence of *P. vivax* was 2.96% (95% $CI_{weighted}$: 2.28, 3.65%). Cluster point prevalences ranged from 0 - 46.15% and presented with wide confidence intervals when using the binomial exact approach (**Supplementary Figure 2**).

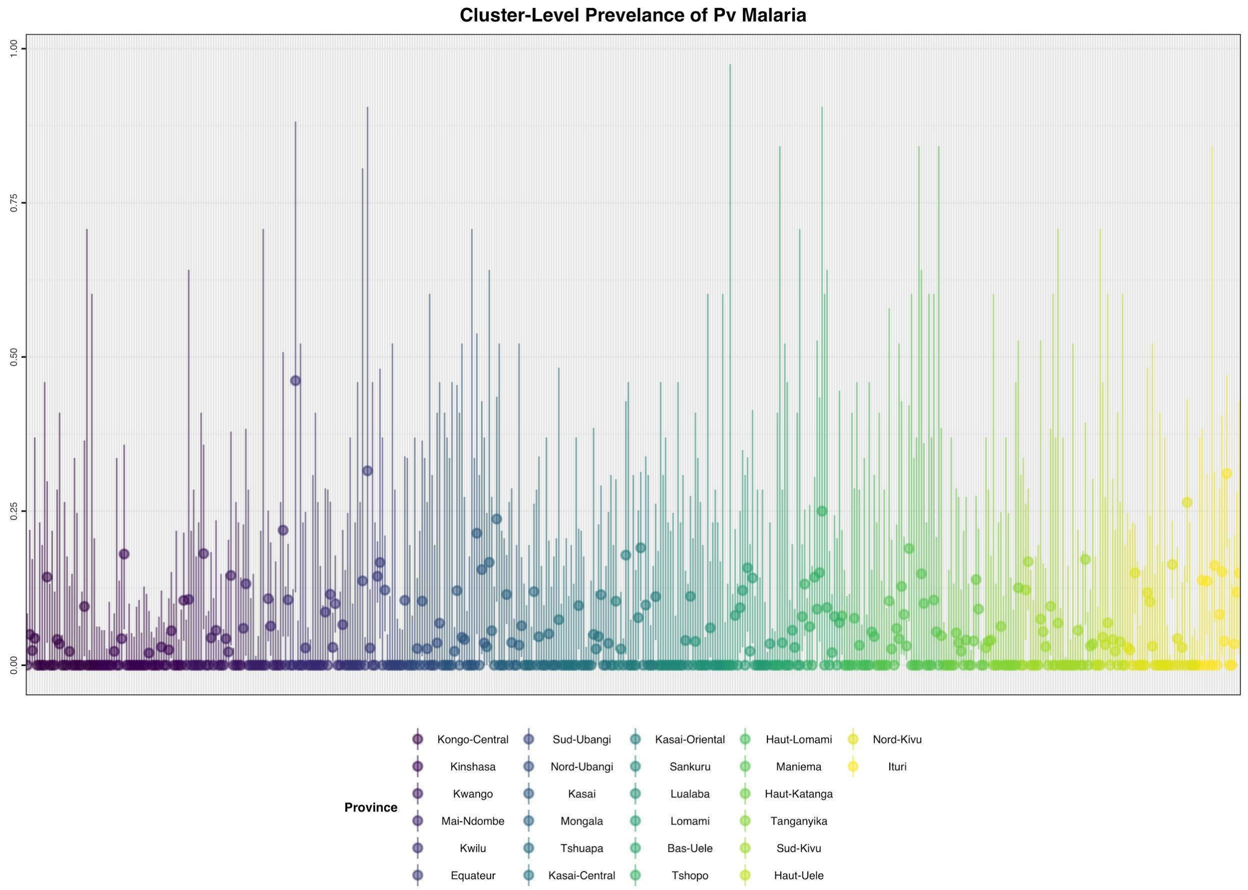

Supplementary Figure 2 - *P. vivax* DHS cluster-level prevalence: The prevalence of *P. vivax* (point) and 95% confidence interval for each DHS cluster are indicated in a purple-yellow spectrum that tracks provinces from east to west-bearings. The 95% confidence intervals were calculated using the binomial exact approach: DHS samplings weights were incorporated by rounding to the nearest integer value.

Covariate Feature Engineering

From the DHS questionnaires, we used data from the household members recode (PR), the HIV testing recode (AR), the geospatial covariate (GC) dataset, and the geographical dataset (GE).[10] Data from the 2013-2014 DRC DHS was downloaded using the `rDHS` package.[11]

In addition to the data provided by the DHS, we downloaded data from several open sources, including: (1) waterways lines and polygon shape-files for the DRC from the Humanitarian OpenStreetMap Team database (https://data.humdata.org/dataset/hotosm_cod_waterways; accessed October 30, 2019); (2)

walking travel times to health care facilities from the Malaria Atlas Project provided at a 0.0083° x 0.0083° spatial resolution[12]; (3) non-human ape (NHA) territories from the International Union for Conservation of Nature Red List database (https://www.iucnredlist.org/; accessed January 21, 2019). Daily daytime air temperature estimates across Africa at a 0.1° x 0.1° spatial resolution were reconstructed from the publicly available Fourier transforms and example R-script from Garske *et al*. 2013[13] while monthly precipitation data with a 0.05° x 0.05° spatial resolution was downloaded from the Climate Hazards Group Infrared Precipitation with Stations (CHIRPS) server using an R-wrapper package (GitHub: `environmentalinformatics-marburg/heavyRain`) for the 2013-2014 DRC DHS study period[14]. Airport locations in the DRC were downloaded from the Humanitarian OpenStreetMap project ((https://data.humdata.org/dataset/ourairports-cod). Airports were restricted to those classified as "medium" or "large". Finally, additional map features included: (1) ocean spatial polygons from Natural Earth (naturalearthdata.com); (2) geographical base-map layers from the Database of Global Administrative Areas (http://www.gadm.org/); (3) country geographies from the R-package, `rnaturalearth`.[15]

Prior to analysis, we identified risk factors for *P. vivax* and *P. falciparum* from a comprehensive literature review.[1,16–18] The relationships among risk factors and our outcome of interest, malaria (i.e. either *P. vivax* or *P. falciparum*) was modeled using a directed acyclic graph (DAG) with the `daggity` graphical user interface and R-package (**Supplementary Figure 3**).[19] As a result, not all risk factors identified were measured and included in the analysis. Although anemia and anti-malarial use were considered to be *a priori* risk factors, both were determined to have cyclic relationships with our outcome of interest, malaria, and were excluded (i.e. anemia and anti-malarial use could not be resolved by the DAG).

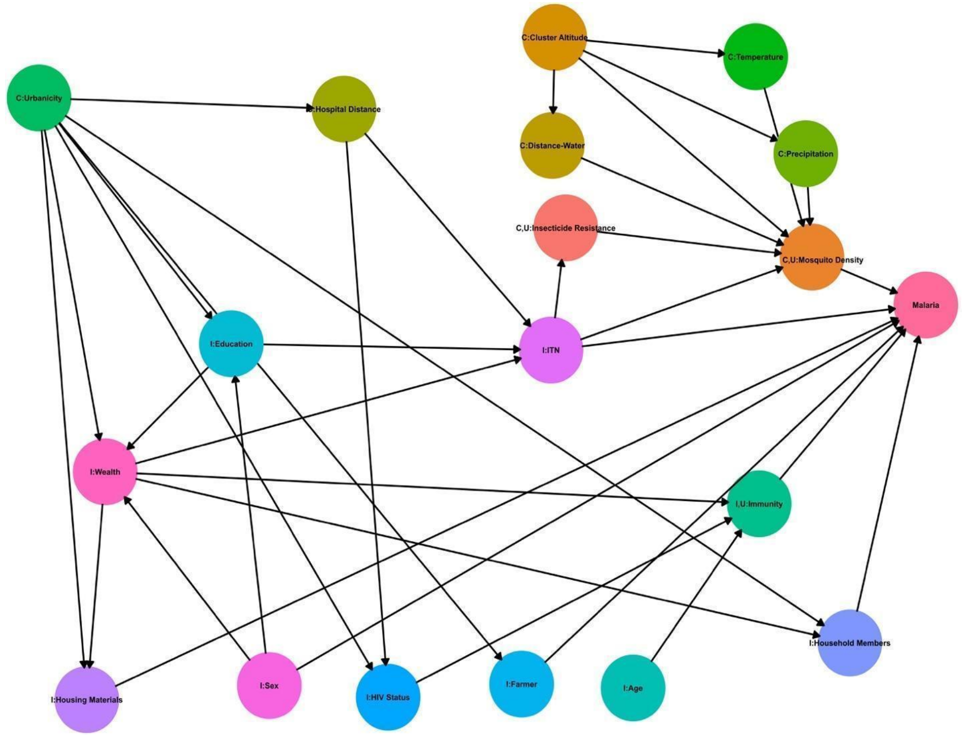

Supplementary Figure 3 - Malaria Risk Factor Directed Acyclic Diagram: Risk factors were identified from an extensive literature search. Similarly, causal relationships among risk factors were based on previous literature and putative associations. Based on our directed acyclic diagram (DAG), we expected urbanicity, cluster altitude, age, and biological sex to all be unconfounded in expectation. Risk factors listed with a "U" were unmeasured, while an "I" and "C" indicates individual and cluster-level covariates, respectively.

Risk factors abstracted from the DHS recodes were mostly kept in their original form, although continuous covariates were standardized. Dichotomized variables were set to have an *a priori* protective referent level. Housing type was coded as either "traditional" or "modern" based on a composite score of floor, wall, and roof type as previously outlined by Tustings *et al.* 2017. We also considered any house that had a metal roof as "modern", given recent findings that metal roofs alone appear to be protective against malaria.[20] Education ascertainment was dichotomized as "lower" (no education; primary) or "higher" (secondary; higher). The farmer risk factor was dichotomized as "farmer" or "not farmer" based on the occupation covariate. Four individuals with "missing" as an occupation were considered "not farmers".

Given that the DHS wealth variable accounts for housing type in its calculation, we recreated the wealth variable in order to avoid issues of collinearity and non-independence between the housing and wealth covariates.[10,18,21] The wealth covariate was recreated based on the instructions by Rustein 2015 and Tustings *et al.* 2017 and using a principal components analysis approach. For a simpler and more straightforward approach, toilet facility, water source, and cooking fuel were categorized as dichotomized variables: positive toilet (flush to piped sewer system, flush to pit latrine, flush to septic tank, flush to somewhere else, pit latrine with slab, ventilated improved pit latrine) versus negative toilet (bucket toilet, hanging toilet/latrine, missing, no facility/bush/field, other, pit latrine without slab/open pit); positive water source (piped into dwelling, piped to yard/plot, piped from the neighbor, tube well or borehole, protected well, bottled water) versus negative water source (public tap/standpipe, unprotected well, protected spring, unprotected spring, river/dam/lake/ponds/stream/canal/irrigation channel, cart with small tank, other, missing); and positive cooking fuel (electricity, biogas, kerosene, coal/lignite, charcoal) versus negative cooking fuel (wood, straw/shrubs/grass, agricultural crop, no food cooked in house, other). The loading values for the first component in the wealth principal component analysis were then used to create a common score. The principal components approach was then repeated for the urban and rural clusters and regressed against the common score to create urban and rural coefficients. As indicated in Rustein 2015, these coefficients were then used to make a composite wealth score and subsequent quintiles of wealth: poorest, poor, middle, rich, and richest. These quintiles were then further dichotomized as poor (poorest, poor) and not poor (middle, rich, richest). Interestingly, in the final composite score, bicycle ownership in urban settings negatively affected the wealth score, while it positively affected the wealth score in rural settings. This may reflect different modes of transportation associated with wealth in different settings or may be a limitation of our analytical approach. In addition, wealth quintiles were slightly unbalanced, again potentially reflecting a limitation of our analytical approach.

We defined insecticide treated net (ITN) usage based on the definition outlined in Tustings *et al.* 2017, which limits the ITN classification to long-lasting insecticidal nets less than or equal to three-years-old at the time of the survey, convention ITNs that were less than or equal to one-year-old at the time of the survey, or any net that was retreated within a year of the survey. All other net-usage was coded as "no net" alongside those individuals that reported not using a net the night prior to the survey.[10]

For each cluster, we took the average walking-travel time to the nearest healthcare facility among all raster squares within 2 km or 10 km radius of the cluster. The 2 versus 10 km boundary depended on the cluster's designation as an urban or rural designation, respectively. This approximates the offsets of geographical coordinates applied by the DHS for each cluster.[10,22] Given that the average-walking travel time and urbanicity were extremely correlated

(*e.g.* points in cities can have zero minutes of travel), we created a new factorized healthcare distance variable with two levels: near or far. A cluster was considered to be "near" to healthcare facilities if it was designated as rural by the DHS and within 1-hour of average walking time or if it was designated as urban by the DHS and within 30-minutes average walking time. This factorization helped to reduce the correlation with urbanicity as well as capture clusters that were more remote from healthcare facilities than may be expected given their urban or rural status.

Distance to water was measured as the minimum greater circle distance between a cluster and a body of water that was either labeled as a "river" or "lake" by the OpenStreetMap water-type. The greater circle distance was log-transformed before being standardized for the risk factor modeling. Greater circle distances were measured using the R `sf` package.[23,24]

Given that the 2013-2014 DRC DHS was conducted in two phases, with the first phase contained to Kinshasa and surrounding regions during months that coincided with the dry-season, while the remaining areas were surveyed during months mostly coinciding with the rainy-season, we elected to take the average daily temperature and monthly precipitation across the dates included in the study (*i.e.* matched months versus matched days for precipitation and temperature, respectively). Although previous studies have shown that lagging precipitation and temperature can improve predictions of malaria transmission in some cases, we felt that we were unable to lag our weather covariates without introducing spatial confounding.[25–29] As a result, for each cluster during each time-period, we first took the average amount of precipitation or daytime temperature among all raster squares within 2 km or 10 km radius of the cluster. As before, the boundary differences reflect the geographic offsets applied by the DHS for each cluster.[10,22] We then aggregated these catchment-area averages for each month into a final study period average. Among the 489 clusters considered, four urban clusters had missing values for precipitation and six urban clusters had missing values for temperature (*N.B.* 4/6 overlapped). For these four missing precipitation clusters, the radius was extended to 6 km while for the six missing temperature clusters the radius was extended to 12 km (reflecting differences in spatial resolution); means were calculated as described above. Precipitation and temperature cluster means were then standardized for the risk factor modeling.

Correlations among risk factors were evaluated using the Szekely-Rizzo-Bakirov distance correlation with the `energy` R package.[30–32] Based on the covariate-pairwise correlations, we determined that covariate collinearity was manageable and no covariates needed to be excluded from the analysis (**Supplementary Figure 4**).

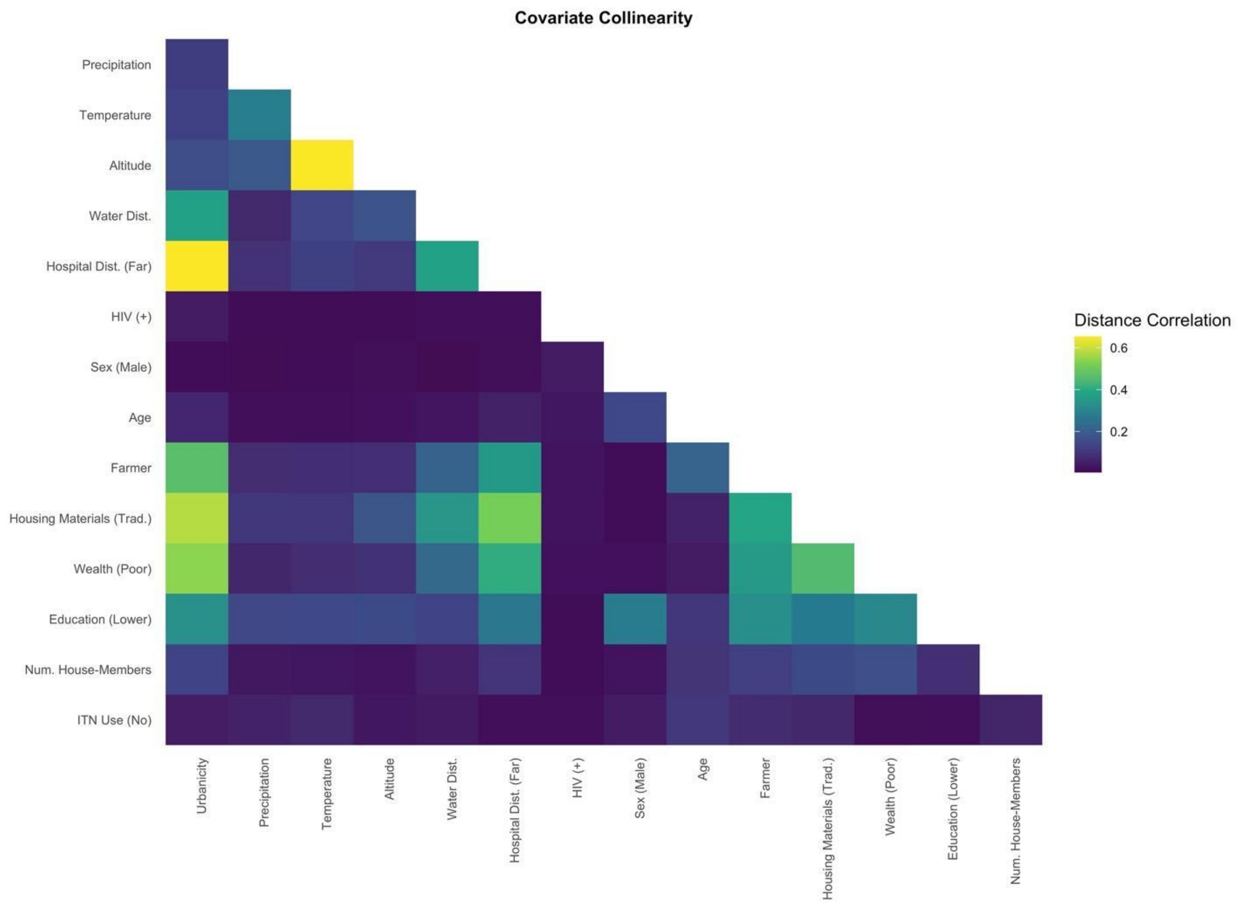

Supplementary Figure 4 - Covariate Collinearity: The correlation between each pair of covariates was explored for potential bias due to extreme collinearity. Although there were strong correlations that were consistent with *a priori* expectations (e.g. wealth and urbanicity), these correlations did not appear to be completely dependent. As a result, all covariates were kept in the analysis. *Abbreviations*: Hospital Dist. – Distance to healthcare facilities, Trad. – traditional, ITN – insecticide treated net, Num. House-Members– Number of Household Members, Water Dist – Distance to water.

P. falciparum-P. vivax Co-infection Model

Interactions between *P. vivax* and *P. falciparum* were examined using an extended version of the independent acquisition of infection model put forth by Akala & Watson *et al.* 2019 to account for individuals that were not infected but still considered in the study population. As in the previous model, we used the observed frequency of each parasite species to fit the expected frequencies of monospecies and cospecies infections using a multinomial likelihood. An additional category -- uninfected -- was added as a parameter to the multinomial model to account for the case when no successful infectious bites occurred. As a result, the unobserved sequence of species, Y that can be passed to a host is now modeled as:

$$Y = \begin{cases} y_1, y_2, , y_k \in S, & \text{if } k > 0 \\ 0, & \text{if } k = 0 \end{cases}$$

Where S was previously defined as the set of *Plasmodium* species of interest and k as the number of infectious bites a host received.[33] Otherwise, the model was unchanged. For the *P. vivax*-*P. falciparum* model, we considered μ as a Poisson distribution and drew 25,000 bootstrap iterations to form the expected infection compositions. Expected infection compositions were then compared against the observed mono- and co-infection data. Overall, mono-infection and co-infection compositions were consistent with the expectation of independent acquisition of parasites, as the observed data fell within the simulated data (**Figure 3A**).

Overlap with Non-Human Ape Permutation Testing

Interactions between NHA territories and *P. vivax* prevalence were assessed using a permutation test with 10,000 iterations. Null distributions for the permutation test were calculated by drawing n_{ape} clusters at random, where n_{ape} was the number of 2013-2014 DRC DHS clusters that overlapped with NHA territories. We then calculated the prevalence of *P. vivax* infections among the selected clusters. We considered NHA territories for (1) *Pan troglodytes* and *Gorilla sp.* and (2) *Pan troglodytes*, *Pan paniscus*, and *Gorilla sp.*, separately, as *P. paniscus* (bonobos) have only recently been shown to harbor *P. vivax*-like parasites at a single field-site (TL2).[34] In contrast, *Pan troglodytes* (chimpanzees) and *Gorilla sp.* have previously been shown to harbor *P. vivax*-like parasites at various prevalences across the DRC.[35] From the permutation tests, NHA territories and *P. vivax* prevalence were not associated ($p > 0.05$). This lack of an association is also evident when visualizing a map of NHA territories and cluster level *P.* vivax prevalences (**Figure 3B**).

Inverse Probability Weights and Prevalence Odds Ratios

The average effect of each risk-factor, A, on our binary outcome of interest Y (i.e. malaria infection), was estimated using marginal structural models (MSMs): $g(P(Y|A = a)) = \beta_0 + \beta_1 a$, where $g(.)$ is a logit link for our prevalence odds ratio effect estimates.[36–39] For each MSM, we adjusted for confounders, L, using inverse probability weights (IPWs).[36–39] IPWs were modeled as $w_i = \frac{1}{f_{A|L}(A|L)}$ for each individual, i in the study population, N. Each weight was stabilized by the marginal mean of the risk factor, such that final weights were: $w_i = \frac{f(A)}{f_{A|L}(A|L)} \text{for} i \in N$. In the case of a binary risk factor, $f_{A|L}(A|L)$ was a probability mass function with each level of A representing the predictive probability of receiving a risk factor given a sequence of confounders. Similarly, in the case of a continuous treatment, $f_{A|L}(A|L)$ was a probability density function with each level of A representing the predictive probability of receiving a dose of the risk factor given a sequence of confounders. In the continuous setting, we assumed that $f(A)$ and $f_{A|L}(A|L)$ followed normal distributions and could be estimated with a standard normal density.[36,38,40]

IPWs were calculated using the super learner algorithm with spatial cross-validation.[41–44] The super learner algorithm was selected for IPW calculations to account for issues of functional form and non-linearity that can bias predictions.[45] We attempted to use a diverse set of candidate algorithms, as the super learner is expected to asymptomatically outperform any individual candidate algorithm as the number of candidate algorithms becomes polynomial in sample size (**Supplementary Table 4**).[41–43] However, weight stability was prioritized over candidate library diversity in 9/11 models.

**Base Learner**	**R-package, Function**	**Relevant Hyperparameters**	**Justification**
Generalized Linear Regression	stat, lm/glm [46]	-	-
Cross-Validated L1/L2 Regularized Regression (x3)	glmnet, cvglmnet [47]	α: 1<br>α: 0.5<br>α: 0	Shrinkage of covariates based on fit
Boosted Generalized Additive Modeling	mboost, gamboost [48]	-	Non-linearity in covariates
K-Nearest Neighbor	kknn, kknn [49]	k: 7<br>Kernel: optimal	Interactions, Non-linearity in Covariates
Single Vector Machines	e1071, svm [50]	Cost: 1<br>Kernel: radial	Interactions, Non-linearity in Covariates
Neural Net	nnet, nnet [51]	Hidden Layers: 1<br>Units in Hidden Layer: 3	Interactions, Non-linearity in Covariates
Random Forest	ranger, ranger [52]	Number of Trees: 500<br>Variables at Node split: √p	Interactions, Non-linearity in Covariates

Supplementary Table 4 - Base Learners used in the Super Learner Algorithm: Various base learners were inputted into the super learner algorithm. The super learner algorithm is an ensemble-based method that optimizes the predictions of base learners using a loss-based approach that minimizes the prediction error. A diverse suite of base learners was selected to account for various non-linear effects as well as interactions among covariates.

Folds for cross-validation were based on K-means clustering of geographical coordinates to account for potential spatial autocorrelation among observations.[44] We selected a K of 15, as it was the inflection point that appeared to minimize the within-cluster sum of squares while avoiding overfitting (**Supplementary Figure 5**).

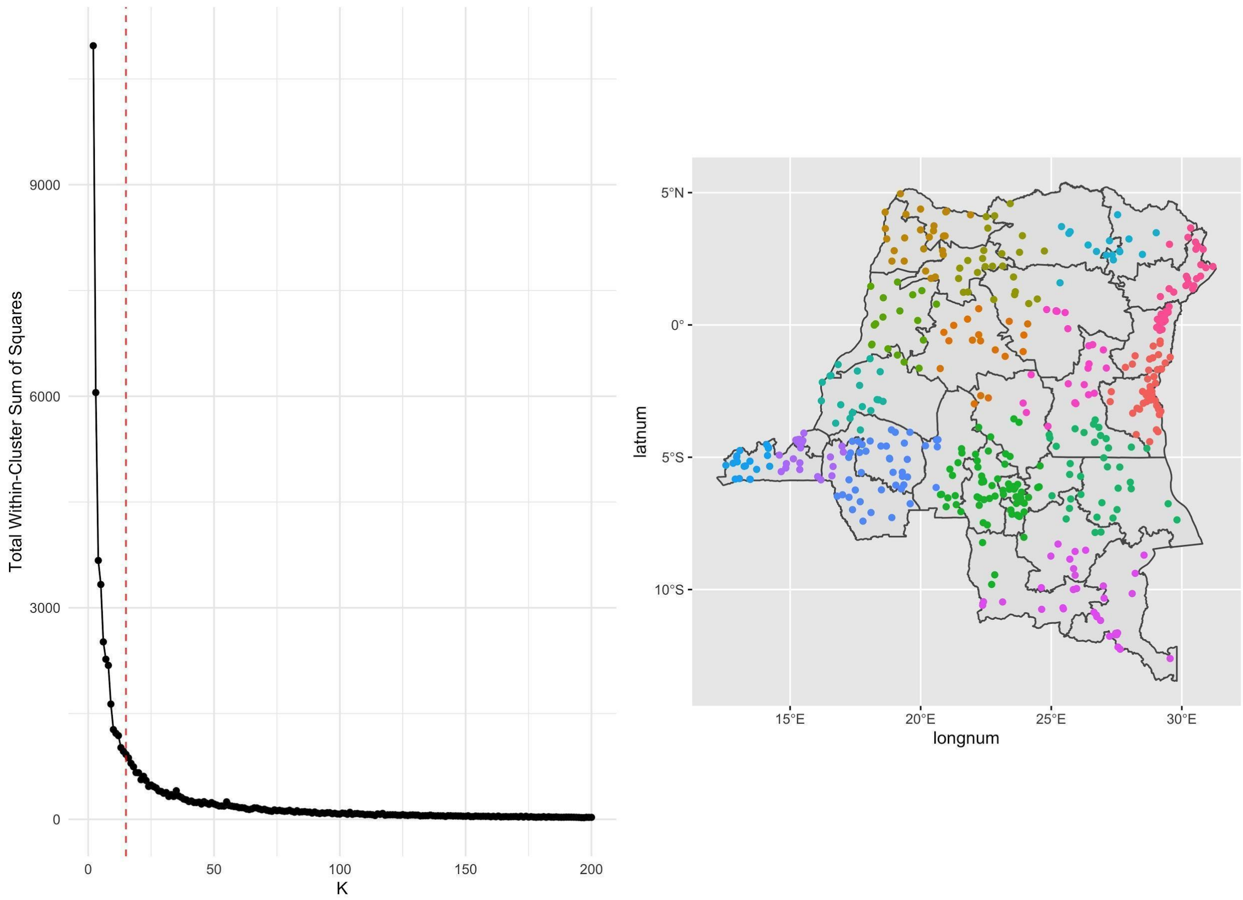

Supplementary Figure 5 - Spatial Cross-Validation K-Clusters: The DRC was partitioned into K-clusters for spatial cross-validation. Based on the geographical K-means total within-cluster sum-of-squares, fifteen clusters appeared to be a reasonable inflection point that did not overfit the data but still captured natural geographic partitions in the DRC (left). The fifteen partitions are mapped to show the geographical partition (right).

All machine-learning models were built and analyzed using the `mlr` package, which provides a machine-learning infrastructure within the R-environment.[53] We assumed that a single iteration of the super learner algorithm was adequate to predict the IPWs. For each risk factor, we considered all covariates that were not on the causal pathway of the risk factor under evaluation as predictors in the IPW-model to account for any "backdoor" paths not considered in our DAG (using the "canonical" set and estimating a "total" effect from `daggity`).[19,36] For risk-factors that were unconfounded in expectation (i.e. biological sex, age, urbanicity, and altitude), no adjustment set was considered (**Supplementary Figure 3**). Weights were incorporated with the R `survey` package and base R functions.[54]

Overall stability of the IPWs were assessed visually and were determined to be relatively stable (**Supplementary Figure 6**; **Supplementary Table 5**). In most cases: 9/11, we found that the IPWs appeared to be more stable or to converge when the "base" model (*i.e.* linear regression or logistic regression for continuous versus categorical variables, respectively; indicated by a 1 in **Supplementary Table 6**) was considered versus the full candidate algorithm library. As a result, these simplified models -- with more stable weights -- were preferred. IPW distributions that are not definitively centered may suffer from lingering issues of structural positivity, residual confounding, or may be correctly identifying multimodal distributions in risk-factor distributions.

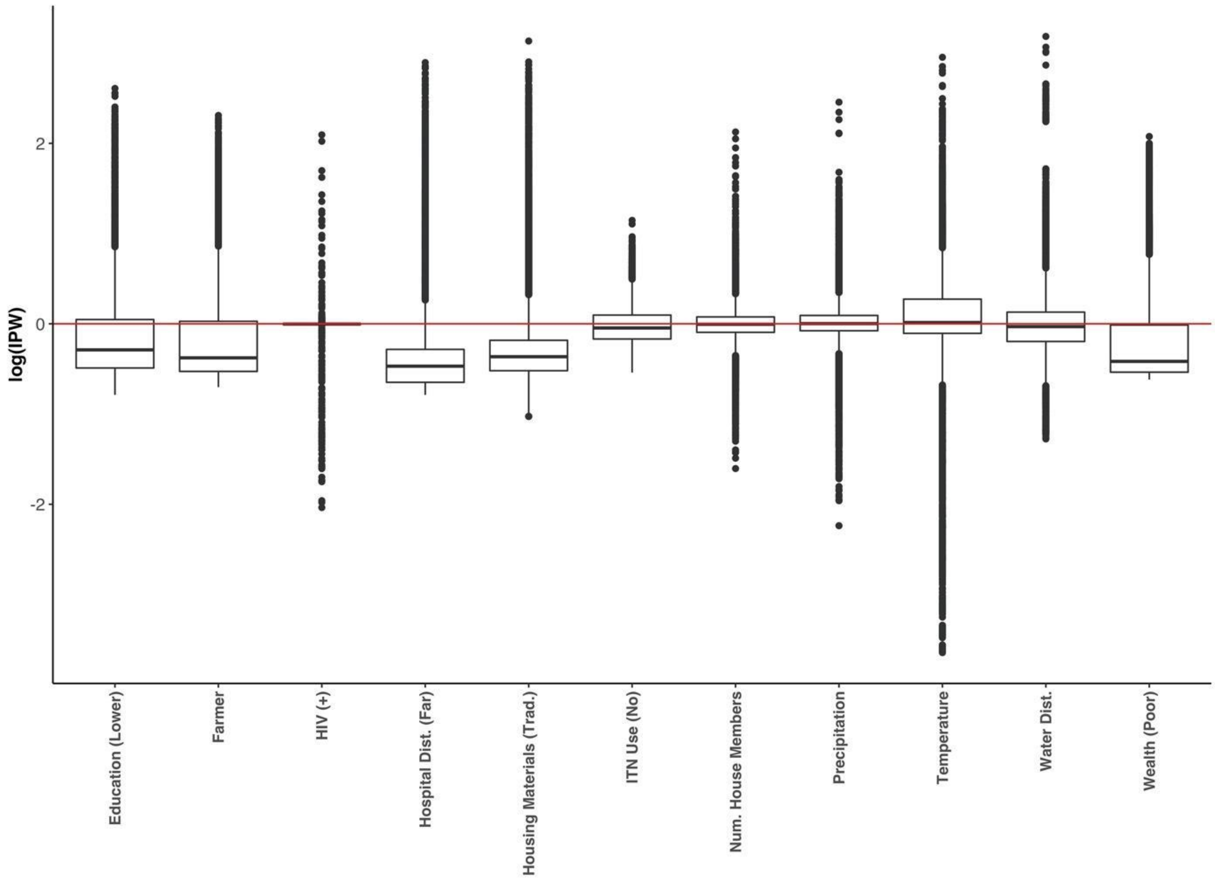

Supplementary Figure 6 - Distribution of Inverse Probability Weights: For each covariate, the distribution of weights for the 15,574 individuals included in the study are shown. Distributions have been log-transformed and appear to be well balanced, although some imbalances persist in distance to health care facilities and housing materials. The boxplot bounds represent the 25th and 75th percentile, while the indicated center is the median. The whiskers are defined as the largest and smallest value up to one-and-a-half times the interquartile range (75th percentile subtracted from 25th percentile), respectively. The dots represent potential outlier points. *Abbreviations*: Hospital Dist. – Distance to healthcare facilities, Trad. – traditional, ITN – insecticide treated net, Num. House-Members– Number of Household Members, Water Dist – Distance to water.

Risk Factor	Min.	Q1	Median	Mean	Q3	Max.
Precipitation	0.11	0.93	1.00	1.05	1.10	11.66
Temperature	0.03	0.90	1.01	1.22	1.31	19.18
Water Dist.	0.28	0.82	0.97	1.10	1.14	24.16
Hospital Dist. (Far)	0.46	0.52	0.63	0.98	0.75	18.09
HIV (+)	0.13	0.99	1.00	1.00	1.00	8.12
Farmer	0.50	0.59	0.69	0.98	1.03	10.07
Housing Materials (Trad.)	0.36	0.59	0.69	1.01	0.83	22.96
Wealth (Poor)	0.54	0.59	0.66	0.98	0.99	7.97
Education (Lower)	0.46	0.61	0.75	1.01	1.05	13.58
Num. House-Members	0.20	0.91	0.99	1.03	1.08	8.38
ITN Use (No)	0.58	0.85	0.95	1.00	1.10	3.15

Supplementary Table 5 - Distribution of Inverse Probability Weights across Covariates: For each covariate, the distribution of weights for the 15,574 individuals included in the study are shown. Distributions are in their original form and appear to be stable with some imbalances appreciated in distance to healthcare facilities and housing materials. Supplementary Figure 6 shows a log-transformed boxplot version of these weights. A*bbreviations*: Hospital Dist. – Distance to healthcare facilities, Trad. – traditional, ITN – insecticide treated net, Num. House-Members– Number of Household Members, Water Dist – Distance to water, Min. -- minimum, Q1 -- first quantile, Q3 -- third quantile, Max. -- maximum.

The effects of the IPW on baseline risk-factor associations (i.e. putative confounding) were assessed using Szekely-Rizzo-Bakirov distance correlations for each risk-factor pair.[30–32] Given that a weight option is not specified in the Szekely-Rizzo-Bakirov distance correlation calculation (energy), we applied our IPWs by sampling observations according to their IPWs. To account for variability in sampling, we created 100 IPW-pseudopopulations for each risk-factor pair. The distribution of pairwise distance correlations for the risk factors was then plotted and compared with no weights applied and with IPWs applied (**Supplementary Figure 7**).

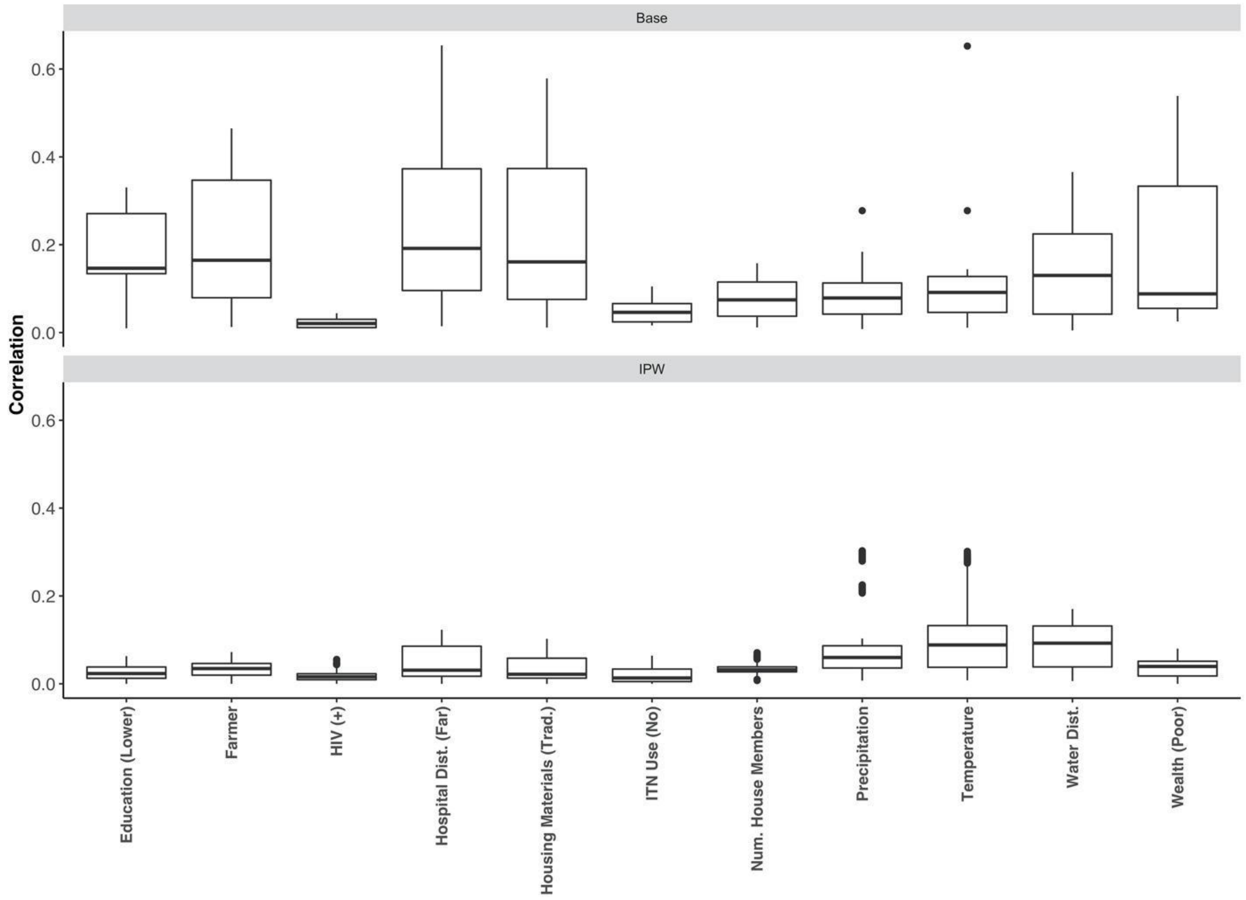

Supplementary Figure 7 - Correlation among Covariates at Baseline and After Application of Inverse Probability Weights: A classic measure of confounding is baseline correlations among covariates, or the unequal distribution of covariates among different treatment classes. Shown for each covariate are the measures of pairwise covariate correlation at baseline (top) and after inverse probability weights (IPWs) have been considered (bottom). The baseline boxplots contain the range of correlations for each risk factor compared to all covariates that were not on the causal pathway (Covariate Sets: Education, n = 10; Farmer, n = 14; HIV, n = 14; Distance to healthcare facilities, n = 12; Housing materials, n = 14; ITN use, n = 14; Number of household members, n = 14; Precipitation, n = 14; Temperature, n = 14; Water Distance, n = 14; Wealth, n = 11). Given that the Szekely-Rizzo-Bakirov distance correlation calculation does not incorporate weights, we used 100 replicates of our IPW to create pseudo-populations and calculate covariate correlations as before. The boxplots demonstrate the range of these 100*n correlation for each risk factor. Baseline covariates show a large degree of correlation -- potentially indicating confounding -- while, for the most part, covariates with IPWs applied show a reduction in pairwise covariate correlations (mean fold-reduction: 3.73, range: 1.10 - 7.37). Interestingly, precipitation and temperature appeared to still have somewhat high pairwise correlations even after applying IPWs. In addition, two risk factors: HIV status and precipitation had maximum IPW-based correlations that exceeded the maximum baseline correlation. The boxplot bounds represent the 25th and 75th percentile, while the indicated center is the median. The whiskers are defined as the largest and smallest value up to one-and-a-half times the interquartile range (75th percentile subtracted from 25th percentile), respectively. The dots represent potential outlier points. *Abbreviations*: Hospital Dist. – Distance to healthcare facilities, Trad. – traditional, ITN – insecticide treated net, Num. House-Members – Number of Household Members, Water Dist – Distance to water.

Covariate	Cross-Validated Risk Coefficient	Base Learner
Precipitation	1	regr
Temperature	1	regr
Water Dist.	0.39	Simple Linear Regression
Water Dist.	0	GLM with Lasso or Elasticnet Regularization (Cross Validated Lambda), α: 1
Water Dist.	0	GLM with Lasso or Elasticnet Regularization (Cross Validated Lambda), α: 0.5
Water Dist.	0.21	GLM with Lasso or Elasticnet Regularization (Cross Validated Lambda), α: 0
Water Dist.	0	Support Vector Machines (libsvm)
Water Dist.	0.01	K-Nearest-Neighbor regression
Water Dist.	0.22	Gradient Boosting with Smooth Components
Water Dist.	0.15	Neural Network
Water Dist.	0.01	Random Forests
HIV (+)	1	regr
Farmer	1	regr
Wealth (Poor)	1	regr
Education (Lower)	1	regr
Num. House Members	0	Simple Linear Regression
Num. House Members	0	GLM with Lasso or Elasticnet Regularization (Cross Validated Lambda), α: 1
Num. House Members	0	GLM with Lasso or Elasticnet Regularization (Cross Validated Lambda), α: 0.5
Num. House Members	0.04	GLM with Lasso or Elasticnet Regularization (Cross Validated Lambda), α: 0
Num. House Members	0.13	Support Vector Machines (libsvm)
Num. House Members	0.03	K-Nearest-Neighbor regression
Num. House Members	0.49	Gradient Boosting with Smooth Components
Num. House Members	0.31	Neural Network
Num. House Members	0	Random Forests
Housing Materials (Trad.)	1	regr
ITN Use (No)	1	regr
Hospital Dist. (Far)	1	regr

Supplementary Table 6 - Cross-Validated Risk and Contribution of Base Learners for each Covariate: Given that the super learner algorithm optimizes the contribution of individual base learners, not all base learners are included in the final predictions for each covariate. In most instances (9/11), the super learner predictions resulted in unstable weights. As a result, we culled the base learner library to either a linear or logistic regression algorithm for continuous and dichotomous covariates, respectively (indicated by a 1 in the Cross-Validated Risk Coefficient column and “regr” in the Base Learner column). *Abbreviations*: Hospital Dist. – Distance to healthcare facilities (near versus far), Trad. – traditional, ITN – insecticide treated net, Num. House Members– Number of Household Members, Water Dist – Distance to water.

Risk Factor	Species	IPTW-pOR	IPTW-pOR, L95	IPTW-pOR, U95	pOR	pOR, L95	pOR, U95
Age	Pv	0.97	0.87	1.07	0.97	0.87	1.07
Altitude	Pv	1.13	0.89	1.44	1.13	0.89	1.44
Education (Lower)	Pv	0.94	0.66	1.32	0.99	0.74	1.34
Farmer	Pv	1.40	0.87	2.27	1.13	0.80	1.60
HIV (+)	Pv	1.01	0.35	2.91	1.86	0.76	4.54
Hospital Dist. (Far)	Pv	2.07	1.24	3.46	1.31	0.82	2.10
Housing Materials (Trad.)	Pv	1.21	0.73	2.00	1.00	0.64	1.57
ITN Use (No)	Pv	0.74	0.53	1.03	0.80	0.58	1.09
Num. House Members	Pv	0.90	0.75	1.09	0.95	0.78	1.14
Precipitation	Pv	0.68	0.52	0.91	0.78	0.63	0.97
Sex (Male)	Pv	1.17	0.89	1.54	1.17	0.89	1.54
Temperature	Pv	0.85	0.65	1.12	0.84	0.64	1.09
Urban	Pv	1.13	0.70	1.82	1.13	0.70	1.82
Water Dist.	Pv	1.01	0.77	1.33	0.97	0.79	1.19
Wealth (Poor)	Pv	0.87	0.57	1.35	0.79	0.55	1.14
Age	Pf	0.81	0.77	0.86	0.81	0.77	0.86
Altitude	Pf	0.73	0.66	0.80	0.73	0.66	0.80
Education (Lower)	Pf	1.43	1.24	1.65	1.18	1.02	1.35
Farmer	Pf	1.17	0.96	1.41	1.20	1.02	1.42
HIV (+)	Pf	0.41	0.15	1.11	0.50	0.26	0.93
Hospital Dist. (Far)	Pf	1.33	1.00	1.77	1.49	1.20	1.85
Housing Materials (Trad.)	Pf	1.36	1.09	1.71	1.84	1.54	2.19

ITN Use (No)	Pf	1.31	1.14	1.50	1.27	1.11	1.45
Num. House Members	Pf	0.97	0.91	1.03	0.97	0.92	1.03
Precipitation	Pf	1.11	0.94	1.31	0.99	0.87	1.12
Sex (Male)	Pf	1.31	1.20	1.43	1.31	1.20	1.43
Temperature	Pf	1.25	1.10	1.42	1.47	1.33	1.63
Urban	Pf	0.70	0.56	0.86	0.70	0.56	0.86
Water Dist.	Pf	0.90	0.76	1.07	1.12	0.99	1.28
Wealth (Poor)	Pf	1.31	1.14	1.51	1.37	1.17	1.61

Supplementary Table 7 - Inverse Probability Weight (IPW) Adjusted and Unadjusted Prevalence Odds Ratios for the Malaria Risk Factors: Inverse probability weight (IPW) adjusted and unadjusted prevalence odd ratios (pOR) risk factor effect estimates for *P. vivax* (Pv) and *P. falciparum* (Pf) are provided with corresponding 95% confidence intervals. IPW adjustments were performed using the super learner algorithm. Unadjusted estimates are modeled using generalized estimating equations with a logit-link and binomial variance accounting for the DHS sample-weights. These bivariate association models are essentially two-by-two tables weighted for the 2013-2014 Demographic Health Survey in the Democratic Republic of the Congo sampling scheme. In instances where the adjusted and unadjusted estimates are the same (age, biological sex, urbanicity, and altitude), the risk factor was expected to be unconfounded at baseline and IPWs were not considered (**Supplementary Figure 3**). *Abbreviations*: Hospital Dist. – Distance to healthcare facilities, Trad. – traditional, ITN – insecticide treated net, Num. House Members – Number of Household Members, Water Dist – Distance to water, Pv – *P. vivax*, Pf– *P. falciparum*.

Spatial and Raster Feature Engineering

In order to incorporate the risk factor covariate information into our spatial models, we downloaded spatial raster data for significant risk factors identified by the MSMs. From these raster surfaces, we then extracted covariate values for each DHS cluster, accounting for the DHS sampling design, and for each DRC province. Specifically, for each cluster and covariate of interest, we took the mean value from all raster squares within a 2 km or 10 km radius with respect to the cluster urban/rural designation.[10,22] For each province, all raster cells within the province boundary were aggregated and summarized as a mean value.

The precipitation raster was used from above, with the surface consisting of mean values over the study period **(Supplementary Materials: Covariate Feature Engineering)**. Values were then standardized as above. Similarly, the previously described raster for the average walking-travel time to the nearest healthcare facility was used as a continuous variable for the spatial model (**Supplementary Materials: Covariate Feature Engineering**). At the cluster-level, the average walking-travel time to the nearest healthcare facility was log-transformed with a 0.5 offset to account for zero-based observations prior to standardization. At the province level, the average walking-travel time to the nearest healthcare facility was simply standardized. In all cases, standardization was performed in favor of model stability.

Bayesian Spatial Prevalence Models

Prevalence maps were fit as mixed generalized linear models with spatially correlated random effects in a Bayesian framework. We modeled prevalence at two different levels: (1) Province-level using the `CARBayes` R-package and (2) Cluster-level using the `PrevMap` R-package.[55,56] DHS sampling weights were accounted for by rounding the number of cases, Y_i, to the nearest whole individual in order to conform with the binomial error distribution of our model. For the province-level models, regions are indexed i, such that n_i is the sample size in the region and p_i is the prevalence in the region. Risk factors that were identified as significant were included as linear predictors, β. As a result, the model was specified as:

$$Y_i|n_i, p_i \sim \text{Binomial}(n_i, p_i)$$
$$logit(p_i) \sim \beta X_i^\intercal + S_i + Z_i$$

Following Lee 2017, for the province-level model, the spatial (S_i) and non-spatial (Z_i) random effects were modeled using a random effect, ϕ with the conditional autoregressive prior proposed in Leroux *et al.* 2000 (hereafter referred to as the Leroux CAR model). Specifically,

$$\beta \sim \text{MultivariateNormal}(\mu_\beta,\ \Sigma_\beta)$$
$$\phi_k|\phi_{-k}, W, \tau^2, \rho \sim \text{Normal}(\frac{\rho\sum_{i=1}^{K} w_{ki}\phi_i}{\rho\sum_{i=1}^{K} w_{ki}+1-\rho},\ \frac{\tau^2}{\rho\sum_{i=1}^{K} w_{ki}+1-\rho})$$
$$\tau^2 \sim \text{InverseGamma}(1,\ 0.01)$$
$$\rho \sim Uniform(0,\ 1)$$

The adjacency matrix, W, was a simple neighborhood matrix, where border sharing was indicated as a binary.[57] Models with the ρ parameter fixed at one assume complete spatial autocorrelation among the random effects (i.e. the Intrinsic CAR or Besag model), while models

the ρ parameter fixed at zero assume independence.[58–60] By allowing ρ to vary under the model, as specified above, we can fit this spatial autocorrelation process.[55,58] Finally, we set the multivariate Gaussian mean prior μ_β as a vector of zeros and the diagonal elements of the covariance matrix, Σ_β, to 100.[55]

For the cluster model, the survey region is the DHS second-level enumeration area, which is a collection of households aggregated at a single set of GPS coordinates (i.e. clusters).[10] In total, there are j total clusters, where $j \in \geq \mathbb{Z}_1$ and clusters (i.e. sampling locations) are indexed as: $i : j$. As a result, the model was specified as:

$$logit(p_i) \sim \beta X_i^\intercal + S_i + Z_i$$

Following the model presented in Giorgi and Diggle 2017, the spatial random effect, S_i, was modeled as a stationary isotropic Gaussian process with variance σ^2 and a Matérn correlation function, $\rho(d; \phi, \kappa)$. Here, d is the distance between any two clusters. Based on an exploratory analysis of the κ that maximized the log-likelihood of our logit-transformed prevalence data, we fixed κ at 0.75. Similarly, Matérn covariance priors and the σ^2 prior were informed by an exploratory linear approximation of the model using a maximum likelihood estimation approach,[56] and were subsequently considered nuisance parameters. The remainder of the model was specified using the following priors:

$$\beta|\sigma^2 \sim \text{Normal}(0, \sigma^2)$$
$$log(\tau^2) \sim \text{Normal}(2,\ 3)$$
$$log(\phi) \sim \text{Normal}(0,\ 1)$$
$$log(\sigma^2) \sim \text{Normal}(-1.75,\ 1.5)$$

The prior for the beta-covariance matrix was assumed to be an identity matrix. Each model was first evaluated with four diagnostic chains using 10,000 burn-in and sampling iterations for the province-level (no thinning) and the cluster-level models (5 thinning). Chains of the fitted parameters were then visually assessed for convergence and appropriate mixing patterns and were required to have a Gelman-Rubin's convergence diagnostic point estimate lower than 1.2[61]. A final long chain with 100,000 burn-in and samping iterations for the province-level model and the cluster-level model were then considered. Thinning was applied in the province-level model and the cluster-level model for every tenth iteration. Chains of the fitted parameters were again visually assessed and all parameters were required to have an effective sample size of at least 500. The effective sampling size was calculated with the `coda` R package while the highest posterior density interval (HPDI) was calculated with the `HDInterval` package for the CarBayes models (the HPDI is provided by `PrevMap`) [62,63].

Predictions were performed using the fitted values for the province level. Similarly, for the cluster level, predictions were made out-of-sample using the fitted covariates under the assumption of a multivariate Gaussian distribution as previously described in Giorgi and Diggle 2017. As above, the precipitation and healthcare distance rasters covariate observations for each of the prediction sampling locations across the DRC at a 0.1° x 0.1° spatial resolution were calculated as the mean value for each raster cell within a 10 km radius (maximum DHS maximum offset).[10,22] As previously described, these covariate values were then transformed for stability. Given that we were performing interpolation, any value in the covariate prediction matrix that exceeded the observed maximum in our fitted covariate matrix was truncated (i.e. the

observed maximum for each covariate served as an upper bound among the predictions to avoid extrapolation; **Supplementary Figure 8**).

Predictions were then calculated for each of the 10,000 thinned sampling iterations for all points in the DRC at a 0.1° x 0.1° spatial resolution. For all risk factors with a spatial component (**Supplementary Materials: Covariate Feature Engineering**), spatial covariates, and spatial models we assumed the WGS84 projection system for simplicity.

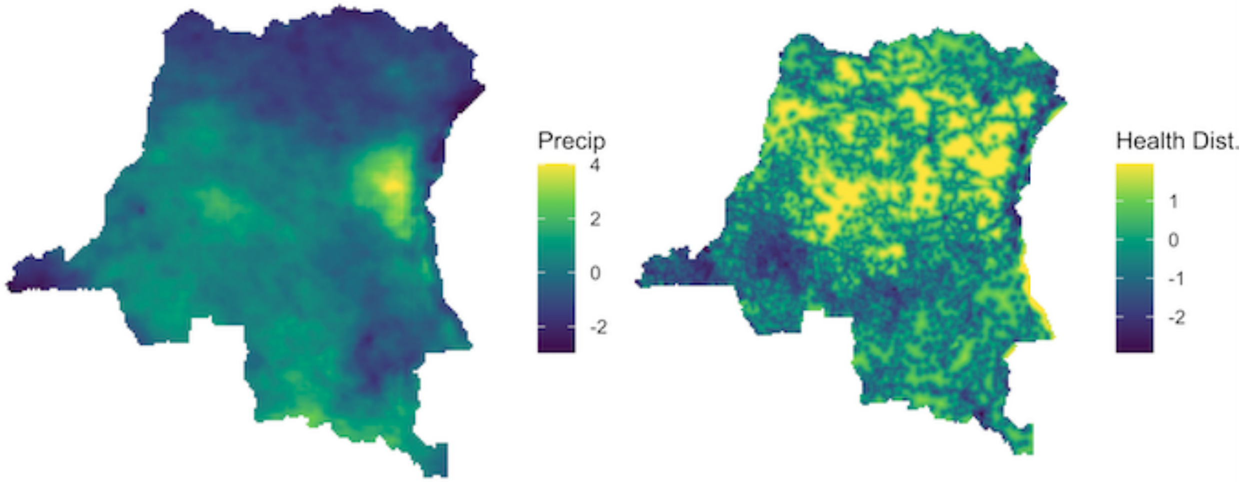

Supplementary Figure 8 - Spatial Raster Prediction Covariates: Spatial covariates that were associated with *P. vivax* infection by the risk factor analysis were included in the spatial prediction prevalence models, and included: precipitation (precip; left) and distance to health care facilities (Health Dist.; right). Values have been transformed as described above.

Level	Parameter	Mean	Median	2.5% HPDI	97.5% HPDI	Effective N
Province	Intercept	-3.57	-3.57	-3.68	-3.46	5,255
	Precip.	-0.03	-0.03	-0.27	0.23	886
	Hosp. Dist.	0.28	0.29	0.03	0.53	795
	τ^2	0.32	0.27	0.07	0.72	3,136
	ρ	0.20	0.13	0.00	0.62	3,433
Cluster	Intercept	-3.63	-3.73	-5.17	-1.65	1,799
	Precip.	-0.06	-0.06	-0.37	0.27	9,500
	Hosp. Dist.	-0.19	-0.18	-0.49	0.09	6,334
	σ^2*	1.99	1.74	0.73	3.87	6,126
	$\phi*$	9.69	7.25	0.01	28.05	2,047
	τ^2*	2.95	2.95	1.81	4.15	1,268

Supplementary Table 8 - Spatial Model Parameter Estimates and Fits: The posterior mean, posterior median, and 95% highest posterior density interval (HPDI) summary statistics are provided for each parameter with respect to the models evaluated. The province-level and cluster-level models included a precipitation covariate and the distance to healthcare facilities (Hosp. Dist) covariate. The effective N is rounded to the nearest integer. The Geweke convergence diagnostic and associated p-value are provided as well. * Considered as nuisance parameters.

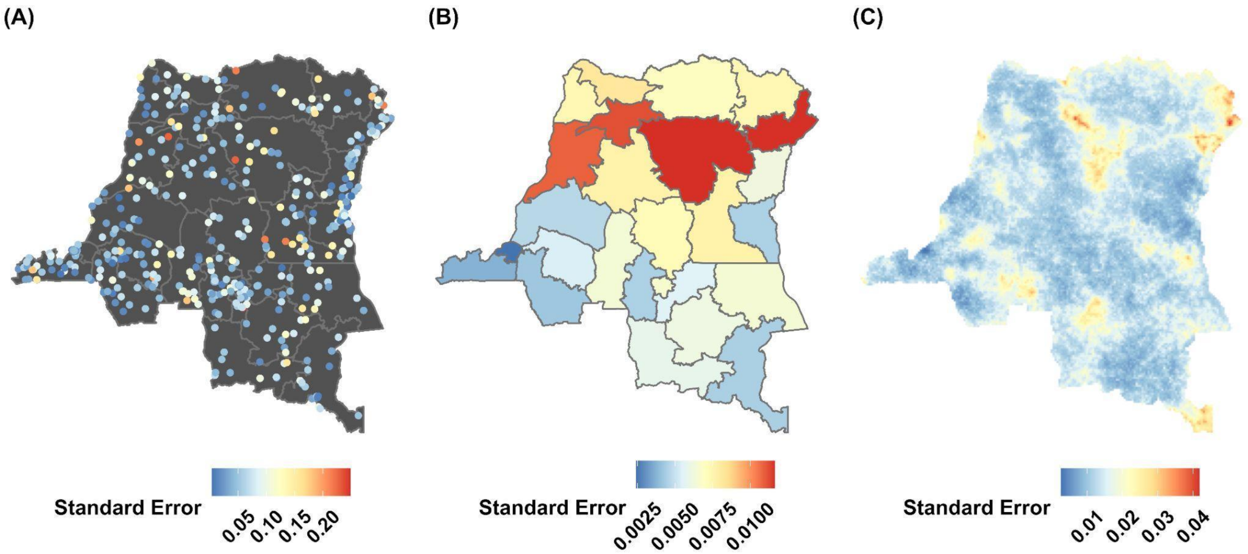

Supplementary Figure 9 - Spatial Model Standard Errors: The standard errors of the posterior prevalence distributions for the point-prevalence cluster estimates (**A**), the province-level model (**B**), the cluster-level model (**C**). For the cluster point prevalence estimates, standard errors were calculated with the binomial exact approach, where DHS samplings weights were incorporated by rounding individuals to the nearest integer. These standard errors are wide reflecting both the low prevalence and low sample size considered. The province-level standard errors ranged from 0.24 - 1.16% and were greatest in the northern and northeastern regions. For the cluster-level model, the standard error ranged from 0.26 - 4.18% with the highest standard errors occurring in regions where the prevalence estimates were greatest in the northeastern and northern regions.

post-hoc Power Calculations

Power calculations were simulated from a population of 15,490 individuals (the weighted N from our study population), where the probability of exposure, $\Pr(E)$ was varied at 10%, 25%, and 50% within the population. For the *P. vivax* models, the overall prevalence of the outcome, O, was set at 3% but was varied in the unexposed group from 0.01 - 3.0% (O_U). In contrast, for *P. falciparum* models, the overall prevalence of the outcome was set at 30% and was varied in the unexposed group from 1.0 - 30.0%. ORs were simulated under the following framework:

$$A_i \sim Bernoulli(\Pr(E)) fori \in 1 : N$$
$$O_E = 2 * O - O_U$$

$$D_i = \begin{cases} Bernoulli(\Pr(O_E)), & \text{if } A_1 = 1 \text{ ,} \\ Bernoulli(\Pr(O_U)), & \text{if}_i = 0 \text{ .} \end{cases}$$

As a result, from the exposure status, A_i, and disease status, D_i, we can calculate the simulated OR using the standard generalized linear model function with a logit-link in R. Power was calculated as the number of iterations that the parameter estimate α was less than 0.05 with respect to each OR.

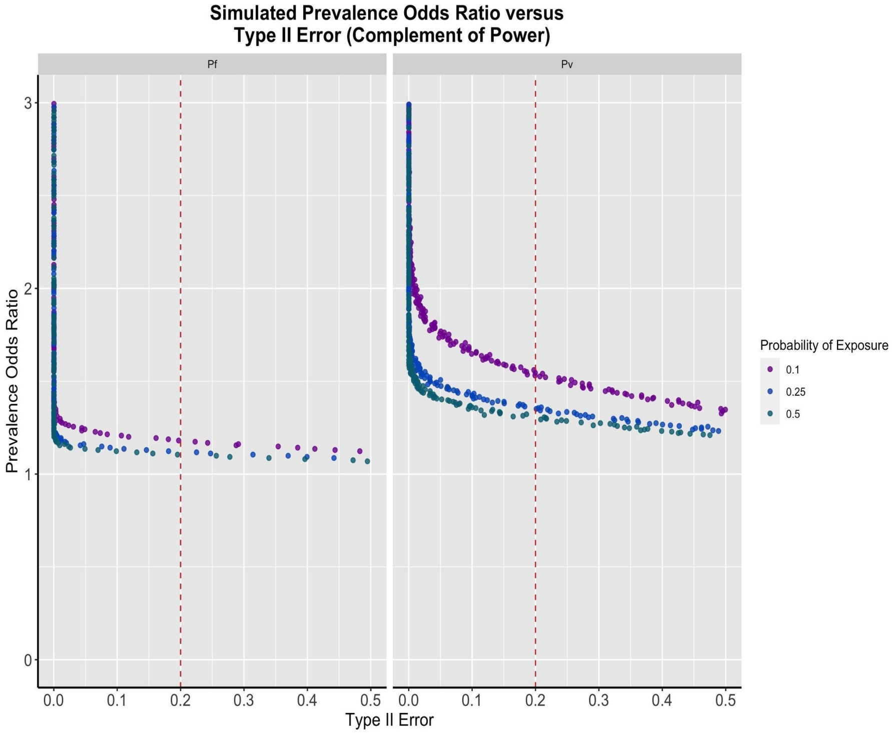

Supplementary Figure 10 - Power Calculations for *P. vivax* and *P. falciparum*: We performed *a posteriori* power calculations to determine the minimum detectable risk factor at varying levels of exposure with 80% power given the prevalence of *P. vivax* and *P. falciparum* identified in our study. At the lowest probability of an individual not being exposed (lowest expected power), we could detect prevalence odds ratio of approximately 1.55 and 1.18 for *P. vivax* ("Pv") and *P. falciparum* ("Pf"), respectively.

Supplementary Note 3: Population Genetics

Publicly Available Whole Genome Sequences

We downloaded 716 publicly available paired-end *P. vivax* or *P. vivax*-like whole genome sequences from across the globe from the European Nucleotide Archive (**Additional File**).[64–79] In addition, *P. cynomologi* paired-end reads were downloaded for the M-strain (Accession: ERS001838).[80]

Alignment, Quality Control, and Variant Discovery

Reads were aligned to the *P. vivax* P01 reference genome (ftp://ftp.sanger.ac.uk/pub/project/pathogens/gff3/CURRENT/PvivaxP01.genome.fasta.gz) with `bwa mem` (v0.7) after undergoing adaptor-trimming with `cutadapt` (v2.3).[81–83] Duplicated reads were discarded and mate-tags were added using `samblaster` (v0.1.24). The quality of the alignments were assessed using the Genome Analysis Toolkit (GATK) `CallableLoci` tool (v3.8-0)[84]. We defined a "callable" loci as sites with greater than or equal to five high-quality reads (MQ >= 10, BQ >= 20). Upon inspection of the DRC isolates, we found that genomic coverage was sparse and only the mitochondrial alignment passed quality-thresholds. As a result, all further analyses were limited to the mitochondria sequence. We then performed short variant discovery using GATK `HaplotypeCaller` followed by joint genotyping across all *P. vivax* samples with GATK `GenotypeGVCFs` (v4.0.3).[85]

For the historical Ebro-1944, we mapped the reads to *P. vivax, P. falciparum*, and human reference genomes using BWA (v0.7.16)[86] and the following options: aln option, 0.01 edit distance, no seed, a penalization of 2 for gap open, and no read trimming; duplicated reads were removed using Picard tools (v2.21.4).[87] To reconstruct the *P. vivax* mtDNA genome, we first removed *P. falciparum* mapping reads from the *P. vivax* mtDNA alignment files using a custom script. Briefly, for each sequence we recorded the edit distance to both the *P. vivax* and *P. falciparum* mtDNA genomes and retained reads with equal or lower edit distance to the *P. vivax* genome. The resulting alignment files were visually inspected with IGV (v1.1) software.[88] Next, to increase resolution, we merged the mtDNA sequences and generated a consensus *P. vivax* mtDNA genome using SAMtools (v1.11)[89] and bcftools (v1.6), restricting to positions with more than two reads and accepting all substitutions that were present in more reads than alternative nucleotides.

Variant Filtering and Consensus Haplotypes

Samples were excluded from downstream processing if less than 95% of their mitochondrial genome was callable (26/720 samples; the three DRC isolates and the *P. cynomologi* M-strain is also included, making 720 samples). Loci were then filtered using a GATK "hard filtering" approach, following previously established guidelines for both single nucleotide variants (SNVs) and insertion-deletions (INDELs).[90] Specifically, we filtered loci with a quality-depth of less than two (QD < 2), position bias (ReadPosRankSum < -8.0 for SNV, ReadPosRankSum < -20.0 for INDELs), strand bias (FS > 60 for SNV, FS > 200 for INDEL, SOR > 3 for SNV, SOR > 10

for INDELs), and low mapping-quality (MQ < 35, MQSR < -12.5) using the GATK `VariantFiltration` and `SelectVariants` tools (v4.0.3).

Following hard-filtering, we performed post-processing of loci and samples using the `vcfR` package and other custom scripts (GitHub: IDEELResearch/vcfRmanip).[91] Passed loci were first limited to biallelic sites. Samples that had more than 5% of loci that had genotyping calls which could not be resolved by GATK were excluded. In addition, samples with more than 15% of SNV genotyped as heterozygous were excluded under an assumption of heteroplasmy. Finally, two samples -- both a part of the *P. vivax*-like Clade 2 from Gilabert *et al.* 2018 -- were found to have a higher-order of diversity than expected (ERS333076, ERS352725) and were subsequently excluded from further analysis. In total, 667 out of 717 samples passed quality thresholds and were included in analyses, which included: 652 globally sourced isolates, our three DRC samples, 11 lab strains, and the *P. cynomologi* M-strain.

Alignment, quality processing, and variant calling pipelines were used with the Python (v3.7.6) Snakemake (v15.3.0) manager. Scripts were based on the pipelines from the Infectious Disease Epidemiology and Ecology Lab (IDEEL) whole genome repository on Github: IDEELresearch/NGS_Align_QC_Pipelines.

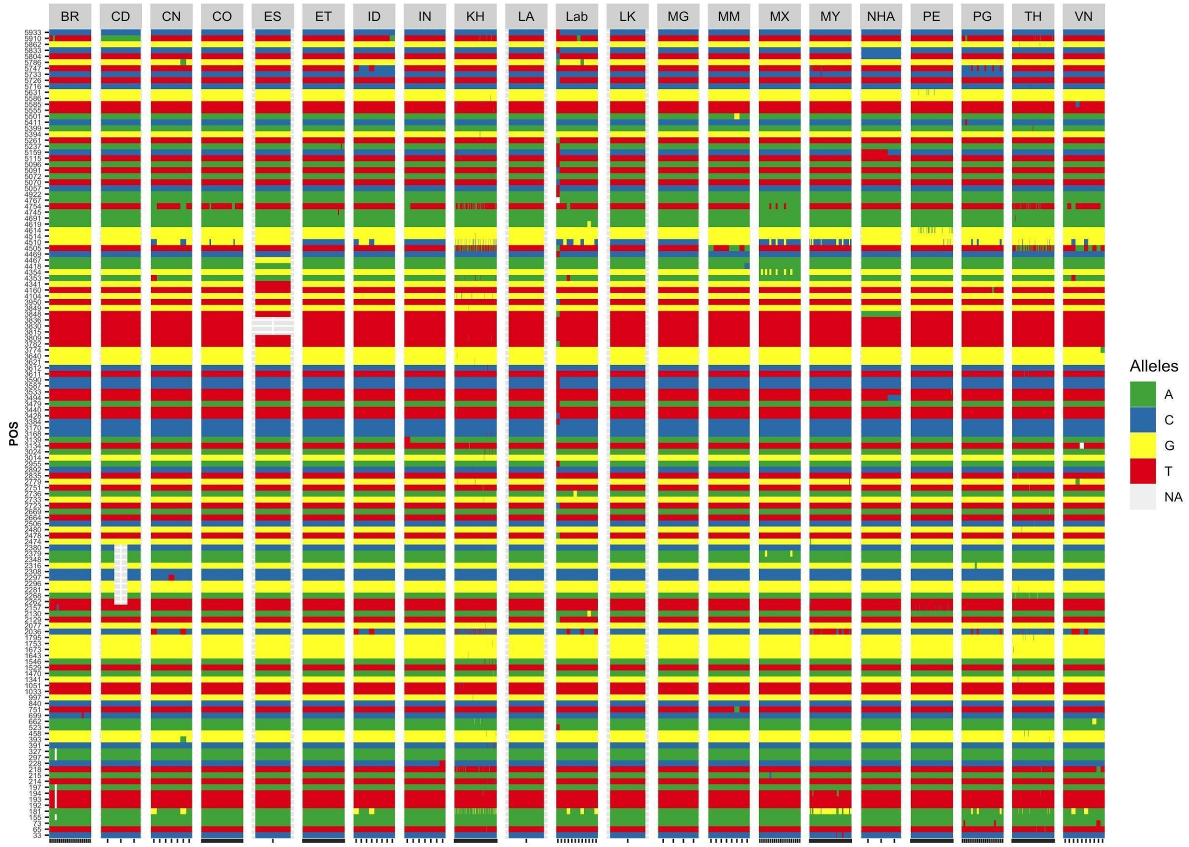

Supplementary Figure 11 - Mitochondrial Genome Consensus Haplotypes: The segregating sites of the 667 *P. vivax* and *P. cynomolgi* consensus haplotypes that passed quality-control (QC) are shown as stacked nucleotide bases (652 globally-sourced isolate consensus haplotypes; 3 DRC consensus haplotypes; 11 lab strain consensus haplotypes, 1 *P. cynomolgi* M-strain consensus haplotype). The *P. cynomolgi* M-strain is included for potential comparison of derived sites, under the assumption that the *P. cynomolgi* loci represent the ancestral allele. *Abbreviations*: Brazil (BR), Democratic Republic of the Congo (CD), China (CN), Colombia (CO), Spain/Ebro1944 (ES), Ethiopia (ET), Indonesia (ID), India (IN), Cambodia (KH), Laos (LA), Sri Lanka (LK), Madagascar (MG), Myanmar (MM), Mexico (MX), Malaysia (MY), non-human apes (NHA), Peru (PE), Papua New Guinea (PG), Thailand (TH), Vietnam (VN), and *P. cynomolgi* M-strain (PcyM).

Population Genetic Statistics and Distances

To explore patterns of diversity among our global isolates, we first measured within-country nucleotide diversity and counts of unique haplotypes using the R-packages, `Biostrings` and `PopGenome` (**Supplementary Table 9**)[92–94]. For the nucleotide diversity calculations, duplicated consensus haplotypes were removed within-countries (*N.B.* non-human apes were considered a "country") and were limited to the DRC and globally sourced *P. vivax* isolates which passed quality controls. Samples with missing genotypes were potentially considered "unique" (*i.e.* a missing loci was considered an "N" loci). This resulted in 142 within-population unique consensus haplotypes (**Supplementary Table 9**).

Similarly, for the genetic distance analysis, only the country-unique consensus haplotypes were considered. Genetic relationships among these country-unique consensus haplotypes isolates was reconstructed using simple base-pair differences (i.e. Hamming's distance) with the `ape` R package [95]. Following this comparison, we selected a representative consensus haplotype from each country, as the consensus haplotype that appeared with the highest frequency (prior to deduplicating) and created a minimum-spanning network with the `poppr` package [96]. For Vietnam, with ten unique consensus haplotypes among ten isolates, a single sequence was randomly selected. Hamming's distance was used to parameterize the minimum-spanning network.

Population	Within-Population Nucleotide Diversity	Within-Population Unique Haplotype Count/QC Passed
Brazil	2.00E-04	5/22
Democratic Republic of the Congo	0	2/3
China	7.79E-04	4/7
Colombia	2.23E-04	3/30
Spain (Ebro1944)	0	1/1
Ethiopia	2.23E-04	3/44
Indonesia	5.57E-04	3/8
India	3.34E-04	3/7
Cambodia	6.47E-04	48/177
Laos	0	1/1
Sri Lanka	0	1/1
Madagascar	0	1/4
Myanmar	3.34E-04	4/8
Mexico	4.01E-04	5/20
Malaysia	5.25E-04	7/52
Non-Human Apes	3.34E-04	2/3
Peru	3.18E-04	7/111
Papua New Guinea	4.77E-04	7/22
Thailand	5.38E-04	25/124
Vietnam	5.97E-04	10/10

Supplementary Table 9 - Within Population Measures of Diversity: For each population, the within-population nucleotide diversity among the country-unique consensus haplotypes and the unique mtDNA consensus haplotype count versus QC-passed strains are presented. Haplotypes with missing data are considered as potentially unique. Overall, there was little within population diversity among the DRC samples, albeit this lack of diversity may be an effect of the small sample size.

Supplementary References

1. Deutsch-Feldman, M. *et al.* Spatial and epidemiological drivers of P. falciparum malaria among adults in the Democratic Republic of the Congo. *BMJ Global Health* (2020) doi:10.1136/bmjgh-2020-002316.
2. Plowe, C. V., Djimde, A., Bouare, M., Doumbo, O. & Wellems, T. E. Pyrimethamine and proguanil resistance-conferring mutations in Plasmodium falciparum dihydrofolate reductase: polymerase chain reaction methods for surveillance in Africa. *Am. J. Trop. Med. Hyg.* **52**, 565–568 (1995).
3. Srisutham, S. *et al.* Four human Plasmodium species quantification using droplet digital PCR. *PLoS One* **12**, e0175771 (2017).
4. Snounou, G. & Singh, B. Nested PCR analysis of Plasmodium parasites. *Methods Mol. Med.* **72**, 189–203 (2002).
5. Mercereau-Puijalon, O., Barale, J.-C. & Bischoff, E. Three multigene families in Plasmodium parasites: facts and questions. *Int. J. Parasitol.* **32**, 1323–1344 (2002).
6. Gruenberg, M. *et al.* Plasmodium vivax molecular diagnostics in community surveys: pitfalls and solutions. *Malar. J.* **17**, 55 (2018).
7. Tanaka, M., Takahahi, J., Hirayama, F. & Tani, Y. High-resolution melting analysis for genotyping Duffy, Kidd and Diego blood group antigens. *Leg. Med.* **13**, 1–6 (2011).
8. Tournamille, C., Colin, Y., Cartron, J. P. & Van Kim, C. L. Disruption of a GATA motif in the Duffy gene promoter abolishes erythroid gene expression in Duffy–negative individuals. *Nat. Genet.* **10**, 224–228 (1995).
9. Ménard, D. *et al.* Plasmodium vivax clinical malaria is commonly observed in Duffy-negative Malagasy people. *Proc. Natl. Acad. Sci. U. S. A.* **107**, 5967–5971 (2010).
10. Croft, T. N., Marshall, A. M. J., Allen, C. K. & Others. Guide to DHS statistics. *Rockville, Maryland, USA: ICF* (2018).
11. API Client and Dataset Management for the Demographic and Health Survey (DHS) Data [R package rdhs version 0.6.3].
12. Weiss, D. J. *et al.* Global maps of travel time to healthcare facilities. *Nat. Med.* **26**, 1835–1838 (2020).
13. Garske, T., Ferguson, N. M. & Ghani, A. C. Estimating air temperature and its influence on malaria transmission across Africa. *PLoS One* **8**, e56487 (2013).
14. Funk, C. *et al.* The climate hazards infrared precipitation with stations—a new environmental record for monitoring extremes. *Scientific Data* **2**, 150066 (2015).
15. South, A. rnaturalearth: World Map Data from Natural Earth. (2017).
16. Taylor, S. M. *et al.* Molecular Malaria Epidemiology: Mapping and Burden Estimates for the Democratic Republic of the Congo, 2007. *PLoS One* **6**, e16420 (2011).
17. Millar, J. *et al.* Detecting local risk factors for residual malaria in northern Ghana using Bayesian model averaging. *Malar. J.* **17**, 343 (2018).
18. Tusting, L. S. *et al.* Housing Improvements and Malaria Risk in Sub-Saharan Africa: A Multi-Country Analysis of Survey Data. *PLoS Med.* **14**, e1002234 (2017).
19. Textor, J., van der Zander, B., Gilthorpe, M. S., Liskiewicz, M. & Ellison, G. T. Robust causal inference using directed acyclic graphs: the R package 'dagitty'. *Int. J. Epidemiol.* **45**, 1887–1894 (2016).
20. Lindsay, S. W. *et al.* Reduced mosquito survival in metal-roof houses may contribute to a decline in malaria transmission in sub-Saharan Africa. *Sci. Rep.* **9**, 7770 (2019).
21. Rutstein, S. O. Steps to constructing the new DHS Wealth Index. *Rockville, MD: ICF International* (2015).
22. Mayala, B., Fish, T. D., Eitelberg, D. & Dontamsetti, T. *The DHS Program Geospatial Covariate Datasets Manual*. (2018).

23. Karney, C. F. F. Algorithms for geodesics. *J. Geodesy* **87**, 43–55 (2013).
24. Pebesma, E. Simple Features for R: Standardized Support for Spatial Vector Data. *The R Journal* vol. 10 439–446 (2018).
25. Darkoh, E. L., Larbi, J. A. & Lawer, E. A. A Weather-Based Prediction Model of Malaria Prevalence in Amenfi West District, Ghana. *Malar. Res. Treat.* **2017**, 7820454 (2017).
26. Ferrão, J. L., Mendes, J. M. & Painho, M. Modelling the influence of climate on malaria occurrence in Chimoio Municipality, Mozambique. *Parasit. Vectors* **10**, 260 (2017).
27. Wangdi, K. *et al.* Development of temporal modelling for forecasting and prediction of malaria infections using time-series and ARIMAX analyses: A case study in endemic districts of Bhutan. *Malar. J.* **9**, 251 (2010).
28. Nkurunziza, H., Gebhardt, A. & Pilz, J. Bayesian modelling of the effect of climate on malaria in Burundi. *Malar. J.* **9**, 114 (2010).
29. Janko, M. M. *et al.* The links between agriculture, Anopheles mosquitoes, and malaria risk in children younger than 5 years in the Democratic Republic of the Congo: a population-based, cross-sectional, spatial study. *The Lancet Planetary Health* **2**, e74–e82 (2018).
30. Székely, G. J., Rizzo, M. L. & Bakirov, N. K. Measuring and testing dependence by correlation of distances. *Ann. Stat.* **35**, 2769–2794 (2007).
31. Székely, G. J. & Rizzo, M. L. Brownian distance covariance. *Ann. Appl. Stat.* **3**, 1236–1265 (2009).
32. Rizzo, M. & Szekely, G. energy: E-Statistics: Multivariate Inference via the Energy of Data. (2019).
33. Akala, H. M. *et al.* Longitudinal characterization of Plasmodium inter-species interactions during a period of increasing prevalence of Plasmodium ovale. *medRxiv* (2020) doi:10.1101/2019.12.28.19015941.
34. Liu, W. *et al.* Wild bonobos host geographically restricted malaria parasites including a putative new Laverania species. *Nat. Commun.* **8**, 1635 (2017).
35. Liu, W. *et al.* African origin of the malaria parasite Plasmodium vivax. *Nat. Commun.* **5**, 3346 (2014).
36. Hernán MA, R. J. M. *Causal Inference*. (Boca Raton: Chapman & Hall/CRC).
37. Hernán, M. A. & Robins, J. M. Estimating causal effects from epidemiological data. *J. Epidemiol. Community Health* **60**, 578–586 (2006).
38. Robins, J. M., Hernán, M. A. & Brumback, B. Marginal structural models and causal inference in epidemiology. *Epidemiology* **11**, 550–560 (2000).
39. Cole, S. R. & Hernán, M. A. Constructing inverse probability weights for marginal structural models. *Am. J. Epidemiol.* **168**, 656–664 (2008).
40. Zhu, Y., Coffman, D. L. & Ghosh, D. A Boosting Algorithm for Estimating Generalized Propensity Scores with Continuous Treatments. *Journal of Causal Inference* vol. 3 (2015).
41. van der Laan, M. J., Polley, E. C. & Hubbard, A. E. Super learner. *Stat. Appl. Genet. Mol. Biol.* **6**, Article25 (2007).
42. Polley, E. C. & van der Laan, M. J. Super Learner In Prediction. (2010).
43. Gruber, S., Logan, R. W., Jarrín, I., Monge, S. & Hernán, M. A. Ensemble learning of inverse probability weights for marginal structural modeling in large observational datasets. *Stat. Med.* **34**, 106–117 (2015).
44. Brenning, A. Spatial cross-validation and bootstrap for the assessment of prediction rules in remote sensing: The R package sperrorest. in *2012 IEEE International Geoscience and Remote Sensing Symposium* 5372–5375 (2012).
45. Pirracchio, R., Petersen, M. L. & van der Laan, M. Improving propensity score estimators' robustness to model misspecification using super learner. *Am. J. Epidemiol.* **181**, 108–119 (2015).
46. R Core Team. R: A Language and Environment for Statistical Computing. (2019).
47. Friedman, J., Hastie, T. & Tibshirani, R. Regularization paths for generalized linear models via coordinate descent. *J. Stat. Softw.* **33**, 1 (2010).
48. Hothorn, T., Buehlmann, P., Kneib, T., Schmid, M. & Hofner, B. mboost: Model-Based Boosting. (2018).

49. Schliep, K. & Hechenbichler, K. kknn: Weighted k-Nearest Neighbors. (2016).
50. Meyer, D., Dimitriadou, E., Hornik, K., Weingessel, A. & Leisch, F. e1071: Misc Functions of the Department of Statistics, Probability Theory Group (Formerly: E1071), TU Wien. (2019).
51. Venables, W. N. & Ripley, B. D. Modern Applied Statistics with S. (2002).
52. Wright, M. N. & Ziegler, A. ranger: A Fast Implementation of Random Forests for High Dimensional Data in C++ and R. *Journal of Statistical Software* vol. 77 1–17 (2017).
53. Bischl, B. *et al.* mlr: Machine Learning in R. *J. Mach. Learn. Res.* **17**, 5938–5942 (2016).
54. Lumley, T. & Others. Analysis of complex survey samples. *J. Stat. Softw.* **9**, 1–19 (2004).
55. Lee, D. CARBayes version 4.6: An R Package for Spatial Areal Unit Modelling with Conditional Autoregressive Priors. *University of Glasgow, Glasgow* (2017).
56. Giorgi, E., Diggle, P. J. & Others. PrevMap: an R package for prevalence mapping. *J. Off. Stat.* (2017).
57. Bivand, R. S., Pebesma, E. & Gómez-Rubio, V. *Applied Spatial Data Analysis with R*. (Springer, New York, NY, 2013).
58. Leroux, B. G., Lei, X. & Breslow, N. Estimation of Disease Rates in Small Areas: A new Mixed Model for Spatial Dependence. in *Statistical Models in Epidemiology, the Environment, and Clinical Trials* 179–191 (Springer New York, 2000).
59. Besag, J., York, J. & Mollié, A. Bayesian image restoration, with two applications in spatial statistics. *Ann. Inst. Stat. Math.* **43**, 1–20 (1991).
60. Lee, D. CARBayes: An R Package for Spatial Areal Unit Modelling with Conditional Autoregressive Priors.
61. Gelman, A. & Rubin, D. B. Inference from Iterative Simulation Using Multiple Sequences. *Stat. Sci.* **7**, 457–472 (1992).
62. Meredith, M. & Kruschke, J. HDInterval: Highest (Posterior) Density Intervals. (2018).
63. Plummer, M., Best, N., Cowles, K. & Vines, K. CODA: Convergence Diagnosis and Output Analysis for MCMC. *R News* vol. 6 7–11 (2006).
64. Parobek, C. M. *et al.* Selective sweep suggests transcriptional regulation may underlie Plasmodium vivax resilience to malaria control measures in Cambodia. *Proc. Natl. Acad. Sci. U. S. A.* **113**, E8096–E8105 (2016).
65. Hupalo, D. N. *et al.* Population genomics studies identify signatures of global dispersal and drug resistance in Plasmodium vivax. *Nat. Genet.* **48**, 953–958 (2016).
66. Loy, D. E. *et al.* Evolutionary history of human Plasmodium vivax revealed by genome-wide analyses of related ape parasites. *Proc. Natl. Acad. Sci. U. S. A.* **115**, E8450–E8459 (2018).
67. Shen, H.-M., Chen, S.-B., Wang, Y. & Chen, J.-H. Whole-genome sequencing of a Plasmodium vivax isolate from the China-Myanmar border area. *Mem. Inst. Oswaldo Cruz* **110**, 814–816 (2015).
68. Shen, H.-M. *et al.* Genome-wide scans for the identification of Plasmodium vivax genes under positive selection. *Malar. J.* **16**, 238 (2017).
69. Gilabert, A. *et al.* Plasmodium vivax-like genome sequences shed new insights into Plasmodium vivax biology and evolution. *PLoS Biol.* **16**, e2006035 (2018).
70. Popovici, J. *et al.* Genomic Analyses Reveal the Common Occurrence and Complexity of Plasmodium vivax Relapses in Cambodia. *MBio* **9**, (2018).
71. Pearson, R. D. *et al.* Genomic analysis of local variation and recent evolution in Plasmodium vivax. *Nat. Genet.* **48**, 959–964 (2016).
72. Auburn, S. *et al.* Genomic analysis of a pre-elimination Malaysian Plasmodium vivax population reveals selective pressures and changing transmission dynamics. *Nat. Commun.* **9**, 2585 (2018).
73. Cowell, A. N., Valdivia, H. O., Bishop, D. K. & Winzeler, E. A. Exploration of Plasmodium vivax transmission dynamics and recurrent infections in the Peruvian Amazon using whole genome sequencing. *Genome Med.* **10**, 52 (2018).
74. Menard, D. *et al.* Whole genome sequencing of field isolates reveals a common duplication of the Duffy binding protein gene in Malagasy Plasmodium vivax strains. *PLoS Negl. Trop. Dis.* **7**, e2489 (2013).

75. Chan, E. R. *et al.* Whole genome sequencing of field isolates provides robust characterization of genetic diversity in Plasmodium vivax. *PLoS Negl. Trop. Dis.* **6**, e1811 (2012).
76. Auburn, S. *et al.* Genomic Analysis of Plasmodium vivax in Southern Ethiopia Reveals Selective Pressures in Multiple Parasite Mechanisms. *J. Infect. Dis.* **220**, 1738–1749 (2019).
77. Ford, A. *et al.* Whole genome sequencing of Plasmodium vivax isolates reveals frequent sequence and structural polymorphisms in erythrocyte binding genes. *PLoS Negl. Trop. Dis.* **14**, e0008234 (2020).
78. Gelabert, P. *et al.* Mitochondrial DNA from the eradicated European Plasmodium vivax and P. falciparum from 70-year-old slides from the Ebro Delta in Spain. *Proc. Natl. Acad. Sci. U. S. A.* **113**, 11495–11500 (2016).
79. van Dorp, L., Gelabert, P., Rieux, A. & de Manuel, M. Plasmodium vivax Malaria viewed through the lens of an eradicated European strain. *bioRxiv* (2019).
80. Pasini, E. M. *et al.* An improvedPlasmodium cynomolgigenome assembly reveals an unexpected methyltransferase gene expansion. *Wellcome Open Res* **2**, 42 (2017).
81. Auburn, S. *et al.* A new Plasmodium vivax reference sequence with improved assembly of the subtelomeres reveals an abundance of pir genes. *Wellcome Open Res* **1**, 4 (2016).
82. Li, H. Aligning sequence reads, clone sequences and assembly contigs with BWA-MEM. *arXiv [q-bio.GN]* (2013).
83. Martin, M. Cutadapt removes adapter sequences from high-throughput sequencing reads. *EMBnet.journal* **17**, 10–12 (2011).
84. Van der Auwera, G. A. *et al.* From FastQ data to high confidence variant calls: the Genome Analysis Toolkit best practices pipeline. *Curr. Protoc. Bioinformatics* **43**, 11.10.1–33 (2013).
85. Poplin, R. *et al.* Scaling accurate genetic variant discovery to tens of thousands of samples. *bioRxiv* 201178 (2017) doi:10.1101/201178.
86. Li, H. & Durbin, R. Fast and accurate short read alignment with Burrows-Wheeler transform. *Bioinformatics* **25**, 1754–1760 (2009).
87. Picard-tools. http://broadinstitute.github.io/picard.
88. Robinson, J. T. *et al.* Integrative genomics viewer. *Nat. Biotechnol.* **29**, 24–26 (2011).
89. Li, H. *et al.* The Sequence Alignment/Map format and SAMtools. *Bioinformatics* **25**, 2078–2079 (2009).
90. Shultz, A. Whole-genome resquencing for population genomics (Fastq to VCF). *Harvard FAS Informatics* https://informatics.fas.harvard.edu/whole-genome-resquencing-for-population-genomics-fastq-to-vcf.html#variantcalling (2018).
91. Knaus, B. J. & Grünwald, N. J. vcfr: a package to manipulate and visualize variant call format data in R. *Mol. Ecol. Resour.* **17**, 44–53 (2017).
92. Pfeifer, B., Wittelsbuerger, U., Ramos-Onsins, S. E. & Lercher, M. J. PopGenome: An Efficient Swiss Army Knife for Population Genomic Analyses in R. *Molecular Biology and Evolution* vol. 31 1929–1936 (2014).
93. Nei, M. Molecular Evolutionary Genetics. (1987) doi:10.7312/nei-92038.
94. Pagès, H., Aboyoun, P., Gentleman, R. & DebRoy, S. Biostrings: Efficient manipulation of biological strings. (2019).
95. Paradis, E. & Schliep, K. ape 5.0: an environment for modern phylogenetics and evolutionary analyses in R. *Bioinformatics* vol. 35 526–528 (2018).
96. Kamvar, Z. N., Tabima, J. F. & Grünwald, N. J. Poppr: an R package for genetic analysis of populations with clonal, partially clonal, and/or sexual reproduction. *PeerJ* **2**, e281 (2014).
