# Supplementary material for: The epidemiology of Plasmodium vivax among adults in the Democratic Republic of the Congo
Source: Nat Commun. 2021 Jul 7;12:4169. doi: 10.1038/s41467-021-24216-3 (PMC8263614; doi:10.1038/s41467-021-24216-3)
Supplement: Supplementary file 2 — Reporting Summary [file 41467_2021_24216_MOESM2_ESM.pdf]

nature research

Corresponding author(s): Brazeau

Last updated by author(s): May 12, 2021

Reporting Summary

Nature Research wishes to improve the reproducibility of the work that we publish. This form provides structure for consistency and transparency in reporting. For further information on Nature Research policies, see our Editorial Policies and the Editorial Policy Checklist.

Statistics

For all statistical analyses, confirm that the following items are present in the figure legend, table legend, main text, or Methods section.

n/a	Confirmed	
☐	☒	The exact sample size (*n*) for each experimental group/condition, given as a discrete number and unit of measurement
☐	☒	A statement on whether measurements were taken from distinct samples or whether the same sample was measured repeatedly
☐	☒	The statistical test(s) used AND whether they are one- or two-sided<br>*Only common tests should be described solely by name; describe more complex techniques in the Methods section.*
☐	☒	A description of all covariates tested
☐	☒	A description of any assumptions or corrections, such as tests of normality and adjustment for multiple comparisons
☐	☒	A full description of the statistical parameters including central tendency (e.g. means) or other basic estimates (e.g. regression coefficient) AND variation (e.g. standard deviation) or associated estimates of uncertainty (e.g. confidence intervals)
☐	☒	For null hypothesis testing, the test statistic (e.g. *F*, *t*, *r*) with confidence intervals, effect sizes, degrees of freedom and *P* value noted<br>*Give P values as exact values whenever suitable.*
☐	☒	For Bayesian analysis, information on the choice of priors and Markov chain Monte Carlo settings
☒	☐	For hierarchical and complex designs, identification of the appropriate level for tests and full reporting of outcomes
☐	☒	Estimates of effect sizes (e.g. Cohen's *d*, Pearson's *r*), indicating how they were calculated

Our web collection on statistics for biologists contains articles on many of the points above.

Software and code

Policy information about availability of computer code

Data collection

Data was downloaded from the Demographic Health Survey and other open source venues. Dried blood spots shipped from the Democratic Republic of the Congo underwent quantitative PCR and nested PCR confirmatory screening.

Data analysis

All scripts and code used to generate these analyses are publicly available on Github (Epidemiological Analyses: nickbrazeau/VivID_Epi; Population Genetic Analyses: nickbrazeau/VivID_Seq). Below are the various packages and code repositories used within these research compendiums.

The R scripts in the VivID_Epi Repository: 03-covar_assoc/01-covar_assoc_energy.R, 06-IPW_ML/02-check_weights_backend_rslurm, 07-spatial_prediction/02-Pv_clst_map_GaussianProc_MCMC_backend, 07-spatial_prediction/03-Pv_get_predictions_backend, and 09-Power/01-PowerCalculation.R were run on the University of North Carolina Longleaf Cluster with the following versions:
R version 3.6.0 (2019-04-26); R-packages:
• tidyverse_1.2.1
• rslurm_0.5.0
• drake_7.13.0
• pwr_1.3-0
• srvyr_0.4.0
• raster_3.4-5
• PrevMap_1.5.3

All other R scripts were ran on a local 2015 MacBook Pro using the following R versions:
R version 4.0.2 (2020-06-22); R-packages
• tidyverse_1.3.0

• raster_3.3-13
• broom_0.7.3
• sp_1.4-5
• elevatr_0.2.0
• ggspatial_1.1.3.9000
• sf_0.9-6
• rdhs_0.7.1
• mlr_2.17.1
• ParamHelpers_1.14
• rvest_0.3.5
• xml2_1.3.2
• srvyr_0.4.0
• survey_4.0
• energy_1.7-7
• mice_3.13.0
• tableone_0.12.0
• RColorBrewer_1.1-2
• spdep_1.1-5
• spData_0.3.8
• geosphere_1.5-10
• environmentalinformatics-marburg/heavyRain
• nickbrazeau/icer (https://github.com/nickbrazeau/icer)
• nickbrazeau/mlrwrapSL (https://github.com/nickbrazeau/mlrwrapSL)
• rgeos_0.5-3
• PrevMap_1.5.3
• CARBayes_5.2
• HDInterval_0.2.2
• coda_0.19-4
• malariaAtlas_1.0.0
• Biostrings_2.58.0
• IDEELResearch/vcfRmanip (https://github.com/IDEELResearch/vcfRmanip)
• seqinr_4.2-5
• vcfR_1.12.0
• ape_5.4-1
• igraph_1.2.5
• poppr_2.8.7
• PopGenome_2.7.5
• glmnet_4.1
• mboost_2.9-4
• kknn_1.3.1
• e1071_1.7-4
• nnet_7.3-15
• ranger_0.12.1

SatScan (v9.6.1)

Bioinformatic Tools (general)
• snakemake (v5.13.0)
• python (v3.7.6)
• pandas (v0.25.1)
• yaml (v5.3.1)
• re (v2.2.1)
• ascp (v3.9.1.168302)
• bwa mem` (v0.7)
• `cutadapt` (v1.16)
• `samblaster` (v0.1.24
• GATK `CallableLoci` tool (v3.8-0)
• GATK `HaplotypeCaller` (v4.0.3)
• GATK `GenotypeGVCFs` (v4.0.3)

Bioinformatic Tools (historical Ebro-1944)
• bwa (v0.7.16)
• Picard tools (v2.21.4)
• SAMtools (v1.11)
• bcftools (v1.6)

For manuscripts utilizing custom algorithms or software that are central to the research but not yet described in published literature, software must be made available to editors and reviewers. We strongly encourage code deposition in a community repository (e.g. GitHub). See the Nature Research guidelines for submitting code & software for further information.

Data

Policy information about availability of data

All manuscripts must include a data availability statement. This statement should provide the following information, where applicable:

- Accession codes, unique identifiers, or web links for publicly available datasets
- A list of figures that have associated raw data
- A description of any restrictions on data availability

Due to data privacy considerations relating to the Demographic Health Survey, the molecular PCR results generated and analyzed during the current study are available from the corresponding author upon reasonable request. All other epidemiological data is publicly available, although the corresponding author can provide intermediary files upon reasonable request. The genomic data produced by the current study is available at the short reads archive bioproject: PRJNA725254.

Field-specific reporting

Please select the one below that is the best fit for your research. If you are not sure, read the appropriate sections before making your selection.

☒ Life sciences ☐ Behavioural & social sciences ☐ Ecological, evolutionary & environmental sciences

For a reference copy of the document with all sections, see nature.com/documents/nr-reporting-summary-flat.pdf

Life sciences study design

All studies must disclose on these points even when the disclosure is negative.

Sample size	This secondary data analysis was conducted on the 2013-2014 Demographic Health Survey (DHS) in the Democratic Republic of the Congo (DRC). The DHS sampling structure involves a first stage, where clusters, or enumeration area, are selected with a known and fixed probability. During the second stage, within each cluster, a subset of households are selected. Finally, among those adults residing in selected households, a subset are consented for HIV and other biomarker testing. To control for this sampling scheme, the DHS weights each individual with an inverse probability weights of selection (sampling weights). The survey was conducted from August 2013 - February 2014. Originally, there were 17,972 survey respondents.
Data exclusions	Of the 17,972 survey respondents with dried blood spots that were successfully shipped to UNC, 13 had incorrectly labeled barcodes. Of the remaining, 17,959 samples, we successfully linked 17,934 to the corresponding PCR results. Among these 17,934 samples, 169 samples had been previously found to fail to amplify human beta-tubulin, which was used as a within-sample positive control, and thus, were excluded. Of these 17,765 samples, 1,402 were missing geospatial data (44 clusters), 237 individuals were not de jure household members, and 535 have sampling-weights set to zero. Finally, 17 observations had missing data and were excluded. This is detailed in the main text (see Figure 1) and the supplementary materials.
Replication	Vivax infections were identified with an initial qPCR assay and then underwent a confirmatory reflex PCR- based assay. This two-step process helps to maximize the specificity of the assay.
Randomization	In order to control for potential confounding in the risk factor analysis, we used inverse probability weights based on our a-priori assumption of covariate-outcome relationship as outlined in our directed acyclic diagram. Given that this was a secondary data-analysis, random allocation was not possible.
Blinding	Given that this was a secondary data-analysis, blinding was not possible.

Reporting for specific materials, systems and methods

We require information from authors about some types of materials, experimental systems and methods used in many studies. Here, indicate whether each material, system or method listed is relevant to your study. If you are not sure if a list item applies to your research, read the appropriate section before selecting a response.

Materials & experimental systems

n/a	Involved in the study	
☒	☐	Antibodies
☒	☐	Eukaryotic cell lines
☒	☐	Palaeontology and archaeology
☒	☐	Animals and other organisms
☐	☒	Human research participants
☒	☐	Clinical data
☒	☐	Dual use research of concern

Methods

n/a	Involved in the study	
☒	☐	ChIP-seq
☒	☐	Flow cytometry
☒	☐	MRI-based neuroimaging

Human research participants

Policy information about studies involving human research participants

Population characteristics	The Demographic Health Survey conducted in the Democratic Republic of the Congo includes men aged 15-59 years and women aged 15-49 years from across the country.
Recruitment	This is a secondary data analysis. Primary recruitment is performed by the Demographic Health Survey.
Ethics oversight	This secondary data analysis was approved the IRBs at the University of North Carolina at Chapel Hill and the Kinshasa School of Public Health.

Note that full information on the approval of the study protocol must also be provided in the manuscript.
